# Arabidopsis LIP5, a Positive Regulator of Multivesicular Body Biogenesis, Is a Critical Target of Pathogen-Responsive MAPK Cascade in Plant Basal Defense

**DOI:** 10.1371/journal.ppat.1004243

**Published:** 2014-07-10

**Authors:** Fei Wang, Yifen Shang, Baofang Fan, Jing-Quan Yu, Zhixiang Chen

**Affiliations:** 1 Department of Botany and Plant Pathology, Purdue University, West Lafayette, Indiana, United States of America; 2 Department of Horticulture, Zijingang Campus, Zhejiang University, Hangzhou, China; University of British Columbia, Canada

## Abstract

Multivesicular bodies (MVBs) play essential roles in many cellular processes. The MVB pathway requires reversible membrane association of the endosomal sorting complexes required for transports (ESCRTs) for sustained protein trafficking. Membrane dissociation of ESCRTs is catalyzed by the AAA ATPase SKD1, which is stimulated by LYST-INTERACTING PROTEIN 5 (LIP5). We report here that LIP5 is a target of pathogen-responsive mitogen-activated protein kinases (MPKs) and plays a critical role in plant basal resistance. Arabidopsis LIP5 interacts with MPK6 and MPK3 and is phosphorylated *in vitro* by activated MPK3 and MPK6 and *in vivo* upon expression of MPK3/6-activating NtMEK2^DD^ and pathogen infection. Disruption of *LIP5* has little effects on flg22-, salicylic acid-induced defense responses but compromises basal resistance to *Pseudomonas syringae*. The critical role of LIP5 in plant basal resistance is dependent on its ability to interact with SKD1. Mutation of MPK phosphorylation sites in LIP5 does not affect interaction with SKD1 but reduces the stability and compromises the ability to complement the *lip5* mutant phenotypes. Using the membrane-selective FM1–43 dye and transmission electron microscopy, we demonstrated that pathogen infection increases formation of both intracellular MVBs and exosome-like paramural vesicles situated between the plasma membrane and the cell wall in a largely LIP5-dependent manner. These results indicate that the MVB pathway is positively regulated by pathogen-responsive MPK3/6 through LIP5 phosphorylation and plays a critical role in plant immune system likely through relocalization of defense-related molecules.

## Author Summary

Pathogen- and stress-responsive mitogen-activated protein kinases 3 and 6 (MPK3/6) cascade plays an important role in plant basal resistance to microbial pathogens. Here we showed that Arabidopsis MPK3 and MPK6 interact with and phosphorylate the LIP5 positive regulator of biogenesis of multivesicular bodies (MVBs), which are unique organelles containing small vesicles in their lumen. Disruption of *LIP5* causes increased susceptibility to the bacterial pathogen *Pseudomonas syringae*. Compromised disease resistance of the *lip5* mutants is associated with competent flg22- and salicylic acid-induced defense responses but compromised accumulation of intracellular MVBs and exosome-like paramural vesicles, which have previously been shown to be involved in the relocalization of defense-related molecules. Phosphorylation by MPK3/6 increases LIP5 stability, which is necessary for pathogen-induced MVB trafficking and basal disease resistance. Based on these results we conclude that the MVB pathway is positively regulated by pathogen-responsive MPK3/6 through LIP5 phosphorylation and plays a critical role in plant immune system probably through involvement in the relocalization of defense-related molecules.

## Introduction

Endosomes traffic molecules from the plasma membrane to intracellular compartments and transport molecules from the biosynthetic apparatus to the sites of action [1], [2]. Several different endosomes have been described based on biochemical composition, morphology, and function. Multivesicular bodies (MVBs) are late endosomes that contain intraluminal vesicles generated when the limiting membrane of the endosome invaginates and buds into its own lumen, thereby allowing cargo-containing intraluminal vesicles to be delivered into and degraded upon fusion with lysosomes or vacuoles [1], [2]. Those proteins retained in the limiting membrane of MVBs, on the other hand, can be delivered to the membrane of lysosomes or vacuoles, or sort back to the plasma membrane or other cellular compartments [1], [2]. Protein sorting into MVBs is highly regulated and is dependent on the action of three distinct protein complexes named ESCRT-I, II and III (Endosomal Sorting Complex Required for Transport) [3]. Ubiquitinated membrane proteins are first recognized by ubiquitin-binding proteins such as the TOM1 families of proteins, which also recruit ESCRT-I components from the cytoplasm. ESCRT-II and ESCRT-III complexes then transiently assembly on the endosomal membrane for cargo sorting, concentration and vesicle formation. For sustained protein trafficking through the MVB pathway, it is necessary that the ESCRT complexes are dissociated and disassembled from the membrane and recycled back into the cytoplasm. The Vps4p/SKD1 AAA ATPase together with its positive regulator Vta1/LIP5 catalyzes the process of ESCRT disassembly in an ATP-dependent reaction [4], [5], [6], [7], [8]. Studies in both yeast and mammalian cells indicate that both Vps4p/SKD1 and Vta1/LIP5 are critical players during MVB biogenesis [5], [9], [10], [11]. In Arabidopsis, disruption of the SKD1 gene is lethal and expression of an ATPase-deficient version of SKD1 causes alterations in the endosomal system and ultimately cell death [12]. Arabidopsis LIP5 interacts strongly with SKD1 and increases *in vitro* the ATPase activity of SKD1 by 4–5 fold [12]. However, disruption of *LIP5* in Arabidopsis causes no phenotypic alterations under normal growth conditions, indicating that the basal levels of the SKD1 ATPase activity are sufficient for plant growth and development [12].

Plants respond to pathogens using two innate immune systems: PTI (pathogen-associated molecular pattern- or PAMP-triggered immunity) and ETI (effector-triggered immunity) [13]. PTI is activated by PAMPs such as bacterial flagellin through mitogen-activated protein kinase(MAPK)-dependent and MAPK-independent signaling pathways (Pitzschke et al., 2009). To suppress PTI, pathogens deliver effectors to plant cells, which may be recognized by plant resistance (R) proteins and activate ETI [13]. ETI is often manifested as hypersensitive responses (HR) associated with rapid programmed cell death [13]. Studies over the past decade have provided increasing evidence for association of vesicle trafficking with plant innate immune systems. In Arabidopsis, pattern-recognition receptor FLS2 confers immunity against bacterial infection through recognition of bacterial flagellin. Following flagellin binding, activated FLS2 undergoes endocytosis and accumulates in late endosomes/MVBs before degradation [14], [15]. Endocytosis of FLS2 functions as a molecular mechanism not only for the attenuation of FLS2 activation but probably also for signaling required for efficient PTI [15], [16], [17]. In addition, N-terminal motifs of a number of NB-LRR R proteins are associated with endomembrane and contribute to disease resistance. Potato R protein R3a relocates from the cytoplasm to late endosomes/MVBs when co-expressed with its cognate effector [18]. Inhibition of the relocalization of R3a to endosomes attenuates the R3a-mediated HR, indicating that relocalization to vesicle in the endocytic pathway is necessary for effector recognition and HR signaling by the R protein [18]. In the penetration resistance of cereal plants against powdery mildew fungal pathogens, which is conferred by local cell wall appositions (papillae), electron or confocal microscopy detected trafficking molecules through late endosomes/MVBs for delivering defense-related materials to papillae, thereby executing a timely and localized defense response to invading pathogens [19], [20], [21], [22], [23]. Similar relocalization of defense-related molecular such as the PENTRATION RESISTANCE 3 (PEN3) ATP binding cassette transporter for cell surface defense in response to conserved pathogen elicitors has also been observed in Arabidopsis [24]. In spite of the extensive microscopic data, genetic analysis of the role of MVBs in plant immune system is not straightforward because mutants for genes essential for MVB biogenesis are often lethal [12], [25]. Previously, the barley GTPase ARFA1b/1c has been localized to MVBs and shown to be important for callose-deposition and penetration resistance of barley [21]. However, the MVB localization of the ARF1 factor was later disputed and evidence was presented for localization of the GTPase to the Golgi and trans-Golgi network (TGN) [26]. Therefore, there is still no compelling genetic evidence for a critical role of the MVB pathway in plant immune system, much less its regulation during plant-pathogen interactions.

MAPK cascade is involved in transduction of pathogen signals to defense responses in plants [27]. In tobacco, Arabidopsis, rice and other plants, stress/pathogen-responsive MAPKs have been identified and extensively studied. In tobacco, WOUND-INDUCED PROTEIN KINASE (WIPK) and SALICYLIC ACID-INDUCED PROTEIN KINASES (SIPK) are activated in resistant tobacco by tobacco mosaic virus and are involved in pathogen-induced HR [28]. In Arabidopsis, functionally redundant MPK3 and MPK6 (orthologs to tobacco WIPK and SIPK) are also responsive to pathogens and pathogen elicitors and functional analyses using both loss- and gain-of-function approaches indicates their critical roles in plant immune responses including PTI, pathogen-induced phytoalexin biosynthesis and stomatal immune responses [27]. Pathogen-responsive MAPKs mediate activation of plant immune responses through phosphorylation of their downstream targets, thereby affecting their activity, stability and other molecular/biochemical properties. In Arabidopsis, MPK3 and MPK6 promote ethylene production through phosphorylation and stabilization of ACS2 and ACS6, two isoforms of the ethylene biosynthetic 1-aminocyclopropane-1-carboxylic acid synthase [29]. MPK3 and MPK6 also phosphorylate WRKY33, a transcription factor important for pathogen-induced expression of camalexin biosynthetic genes [30]. MPK3 and MPK6 also regulate Arabidopsis defense gene expression and disease resistance through phosphorylation of ethylene response factors [31], [32]. Other substrates of MPK3 and MPK6 have also been identified using a variety of approaches including proteomic and bioinformatics procedures but only a few of them have been functionally analyzed [33], [34], [35], [36]. However, our knowledge about the molecular mechanisms underlying the important biological functions of MPK3/MPK6 in plant immune responses is still limited.

In this study, we report identification of Arabidopsis LIP5, a positive regulator of SKD1 AAA ATPase of MVB biogenesis, as an interacting protein and a substrate of pathogen-responsive MPK6/MPK3. Functional analysis with *lip5* T-DNA insertion mutants indicates that LIP5 plays a critical role in pathogen-induced MVB trafficking and in basal resistance to *Pseudomonas syringae* strains. The critical role of LIP5 in plant immune system is dependent on its ability to interact with SKD1. Further analysis reveal that LIP5 is expressed at low levels in healthy plants but its protein levels can be substantially elevated through phosphorylation by the pathogen-responsive MPK cascade. Mutation of MPK phosphorylation sites in LIP5 does not affect its interaction with SKD1 but reduces its stability and, as a result, compromises its ability to complement the basal resistance of the *lip5* mutant plants. These results provide genetic evidence for a critical role of induced MVB biogenesis in plant basal resistance and establish an important mechanism for the regulation of vesicle trafficking during plant-pathogen interactions.

## Results

### Pathogen-Responsive MAPKs Interacts with LIP5

**Figure 1:**
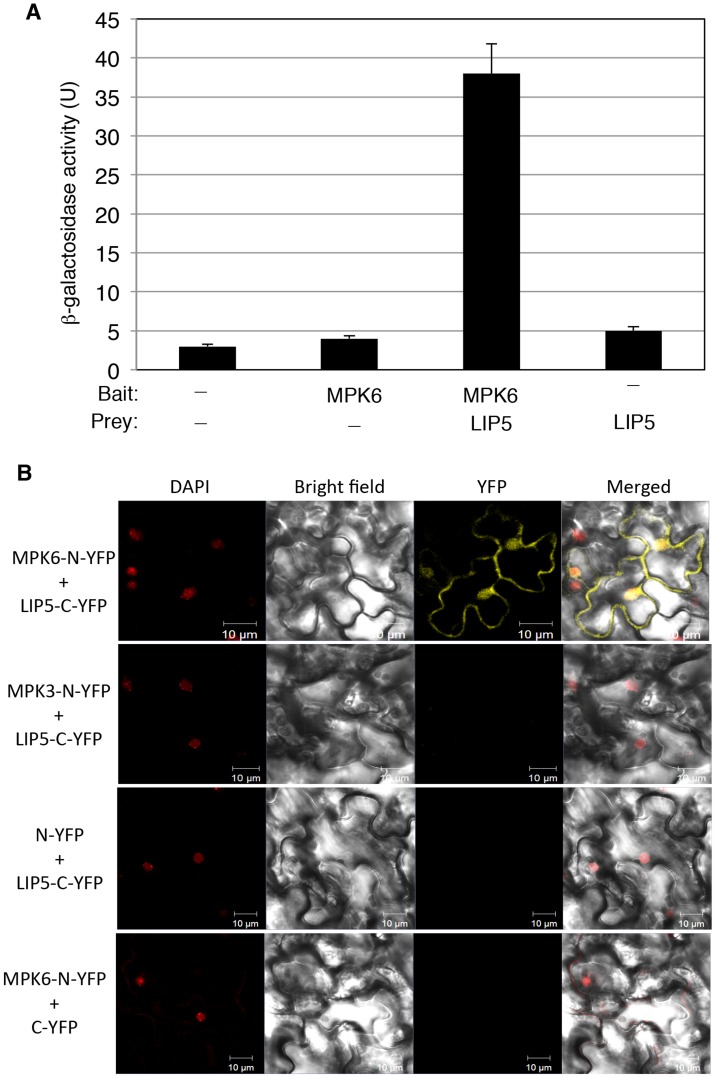
Interaction of MPK6 with LIP5. (**A**) Yeast two-hybrid assays of MPK6 interaction with LIP5. Full-length *MPK6* and *LIP5* coding sequences were introduced into the pBD-Gal4 bait vector and pAD-Gal4 prey vector, respectively. The fusion bait and prey constructs were cotransformed into yeast cells. Empty pBD-Gal4 and pAD-Gal4 vectors were used as negative controls (−). Yeast transformants were analyzed for *LacZ* reporter gene expression through assays of β-galactosidase activity using ONPG as a substrate. Five separate colonies per construct were used for assays of LacZ β-galactosidase activity. (**B**) BiFC analysis of MPK6 interaction with LIP5. Fluorescence was observed in both the cytosolic and nuclear compartments of Arabidopsis leaf epidermal cells, which results from complementation of the N-terminal part of the YFP fused with MPK6 (MPK6-N-YFP) with the C-terminal part of the YFP fused with LIP5 (LIP5-C-YFP). Little or no fluorescence was observed when MPK3-N-YFP was coexpressed with LIP5-C-YFP, when MPK6-N-YFP was coexpressed with C-YFP or when N-YFP was coexpressed with LIP5-C-YFP. DAPI staining, bright-field images, YFP epifluorescence images and overlay images of the same cells are shown. Bars = 10 µm.

Pathogen-responsive MPK3 and MPK6 positively regulate pathogen-induced expression of *WRKY33*, which encodes a WRKY transcription factor important for plant resistance to necrotrophic pathogens and pathogen-induced phytoalexin biosynthesis [30]. MPK3 and MPK6 activate WRKY33 through phosphorylation and activated WRKY33 recognizes its own promoter and activates its own expression [30]. Without realizing the positive feedback mechanism of *WRKY33* induction by MPK3/6, we were initially interested in identifying substrates of the pathogen-responsive MAPKs that may mediate pathogen induction of *WRKY33*. To identify possible substrates of the MAPKs, we cloned MPK3 and MPK6 full-length coding sequence (CDS) in frame into pBD-GAL4 plasmid and used them as baits for yeast two-hybrid screens. After screening 2×10^6^ independent transformants of an Arabidopsis cDNA prey library, we identified positive clones by prototrophy for His and by *LacZ* reporter gene expression through assays of β-galactosidase activity. One of the positive clones identified with MPK6 as bait encodes LYST-INTERACTING PROTEIN 5 (LIP5, At4g26750). As the clone identified from the library screening contains only the 3′ terminal part of *LIP5*, we cloned its full-length coding sequence (CDS) into pAD-GAL4 to generate the pAD-LIP5 fusion construct and retested the interaction in yeast. Yeast cells co-transformed by pBD-MPK6 and pAD-LIP5 were able to grow on -His selective media (data not shown) and were positive for *LacZ* reporter gene expression based on the β-glucosidases activity (Figure 1A).

To determine whether MPK6 and LIP5 interact *in vivo*, we conducted BiFC (bimolecular fluorescence complementation) in transgenic Arabidopsis plants. We fused MPK6 and LIP5 to the N- and C-terminal yellow fluorescent protein (YFP) fragments to generate MPK6-N-YFP and LIP5-C-YFP fusion constructs, respectively. The fusion constructs under control of the CaMV *35S* promoter were transformed into Arabidopsis plants and positive transformants were identified by RNA blotting and crossed to generate transgenic lines that co-expressed MPK6-N-YFP and LIP5-C-YFP. In these co-expressing transgenic lines, BiFC signals were detected in both the cytoplasm and the nucleus (Figure 1B). Control lines in which MPK6-N-YFP was co-expressed with unfused C-YFP or unfused N-YFP was co-expressed with LIP5-C-YFP did not show BiFC signals (Figure 1B).

No positive LIP5 clones were identified from our yeast two-hybrid screens with MPK3 as bait. However, in a published protein-protein-interaction map of Arabidopsis generated by testing all pairwise combinations of a collection of approximately 8,000 Arabidopsis open reading frames with an improved high-throughput binary interactome mapping pipeline based on the yeast two-hybrid system, one of the interacting partners of MPK3 is LIP5 [37]. Using both yeast two-hybrid assays and BiFC, however, we found that the interaction, if any, between MPK3 and LIP5, was much weaker than that between MPK6 and LIP5 (Figure 1B). The discrepancy could be caused by the transient nature of the interaction between LIP5 and MPK3 or the less accessible interaction domain of the MPK3 fusion proteins in our yeast two-hybrid assays and BiFC. As will be described later, both *in vitro* and in-gel assays showed that LIP5 could be phosphorylated not only by MPK6 but also by MPK3. Based on these results, we conclude that LIP5 is capable of interacting with MPK6 and, to a less extent, with MPK3.

### Hyper-susceptibility of *lip5* Mutants to *P. syringae*

Signaling through MPK3/6 in Arabidopsis and their orthologs in other plants plays critical roles in plant immune system [27]. As an interacting partner and potential substrate of the pathogen-responsive MAPKs, LIP5 may act downstream of the MAPKs in plant responses to pathogens. To determine the role of LIP5 directly, we characterized two T-DNA insertion mutants for *LIP5*. The *lip5-1* null mutant (SAIL_854_F08), which contains a T-DNA insertion in the last exon of *LIP5* (see Figure S1A), has been previously isolated and characterized with no apparent phenotype under normal growth conditions [12]. The *lip5-2* mutant (GABI_351F05) contains a T-DNA insertion in the last intron (see Figure S1A) and also appears to be null based on qRT-PCR (see Figure S1B). Although there was no major phenotype in plant morphology throughout their entire life cycle, the growth of both *lip5-1* and *lip5-2* mutants under our normal growth conditions were slightly slower and their leaves were slightly paler green and flatter than those of wild-type plants (see Figure S1C). We also observed that the seed yields of both *lip5-1* and *lip5-2* mutants were about 70% of those of wild-type plants under normal growth conditions.

**Figure 2:**
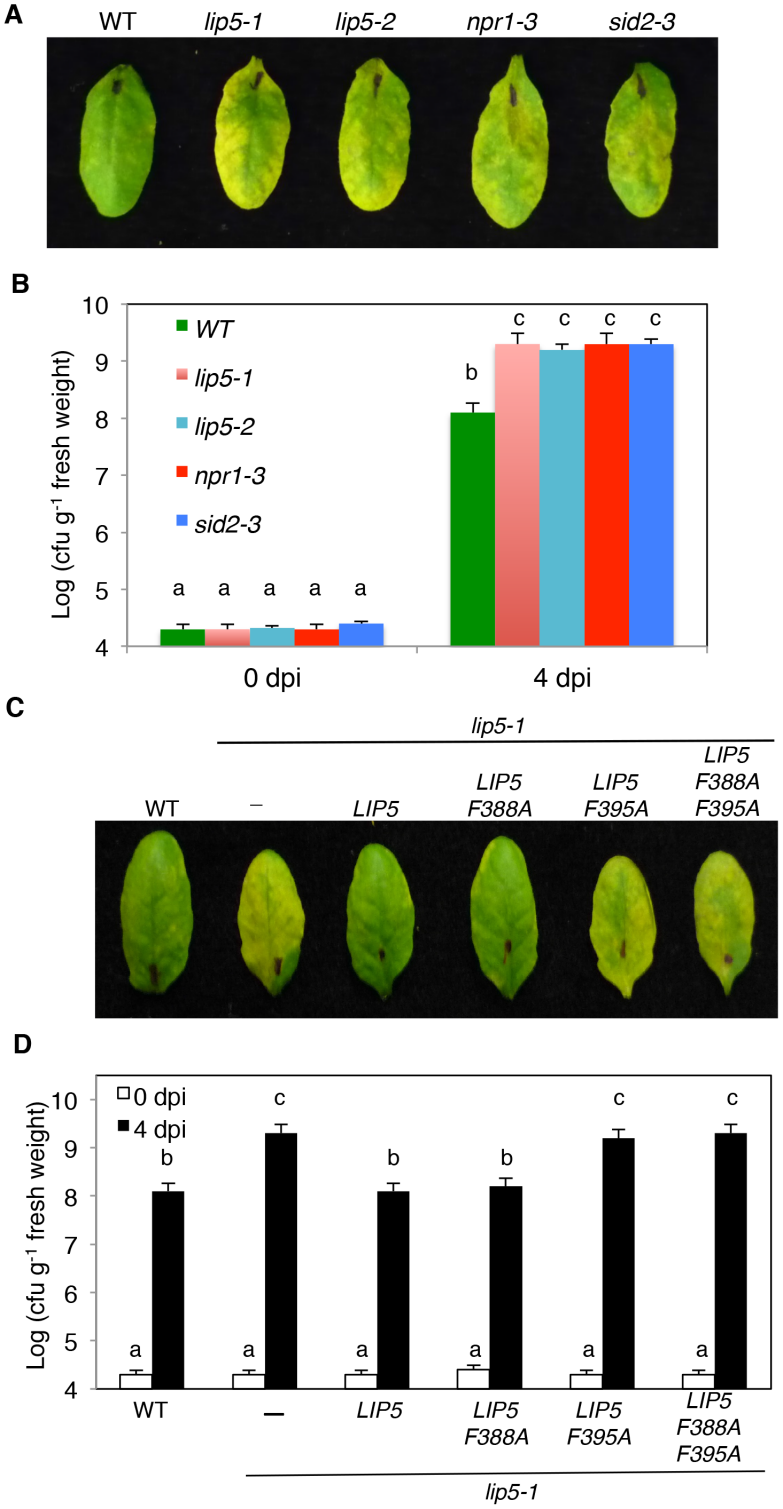
Analysis of *lip5* mutants for responses to a virulent *Pst*DC3000 strain. (**A**) Disease symptom development in the *lip5*, *sid2* and *npr1* mutants. Wild type (WT) and mutant plants were infiltrated with a suspension of *Pst*DC3000 (OD_600_ = 0.0002 in 10 mM MgCl_2_). (**B**) Increased bacterial growth in the *lip5*, *sid2* and *npr1* mutants. Pathogen inoculation of wild-type and mutant plants was performed as in **A**. Samples were taken at 0 or 4 dpi to determine the bacterial growth. (**C**) Disease symptom development in complemented *lip5* mutant plants. WT and *lip5-1* mutant plants complemented with an empty vector (−) or with various *LIP5* genes were infiltrated *Pst*DC3000 as in **A**. (**D**) Bacterial growth in complemented *lip5* mutant plants. Pathogen inoculation and determination of bacterial growth were performed as in **B**. Pictures of representative inoculated leaves in **A** and **C** were taken at 4 dpi. The means and standard errors in **B** and **D** were calculated from 10 plants for each mutant. According to Duncan's multiple range test (P = 0.05), means of colony-forming units (cfu) do not differ if they are indicated with the same letter.

To test possible change in plant disease resistance, we first compared the *lip5* mutants with Col-0 wild-type plants for response to the virulent *P. syringae* pv. *tomato* strain DC3000 (*Pst*DC3000). As controls, we also included *sid2-3* and *npr1-3* mutants, which are deficient in SA biosynthesis and signaling, respectively [38], [39]. When inoculated with the virulent bacterial pathogen, *lip5* mutants developed severe chlorosis based on both visual appearance (Figure 2A) and chlorophyll levels (see Figure S2A) as observed in the *sid2* and *npr1* mutants, while wild-type plants displayed only very mild disease symptoms at 3–4 days post inoculation (dpi). Furthermore, the levels of the growth of the virulent bacterial pathogen in the *lip5*, *sid2* and *npr1* mutants were about 10–20 times higher than those in the wild-type plants (Figure 2B). Thus the *lip5* mutants were as susceptible to the virulent bacterial pathogen as *sid2* and *npr1* mutants.

We also compared wild type and *lip5* mutant plants for responses to avirulent strains of the bacterial pathogen. We first tested the plants for HR development after inoculation with a high dose (OD_600_ = 0.1) of *Pst*DC3000 carrying *avrRpm1*, *avrB* or *avrRpt2*. Visible tissue collapse and cell death were already developed in wild-type leaves inoculated with *Pst*DC3000(*avrRpm1*), *Pst*DC3000(*avrB*) or *Pst*DC3000(*avrRpt2*) by 6 hours post inoculation (hpi) (see Figure S3A). The *lip5-1* mutant leaves were normal in HR development after infiltration with *Pst*DC3000(*avrRpm1*) or *Pst*DC3000(*avrB*) (see Figure S3). However, no visible tissue collapse was developed in the *lip5-1* mutant plants infiltrated with *Pst*DC3000(*avrRpt2*) at 6 hpi as in wild-type leaves, although the mutants had well-developed HR at 24 hpi (see Figure S3A). Thus, HR development induced by one of the three tested avirulent strains of *Pst*DC3000 was delayed, but not abolished in the *lip5-1* mutant plants. Similar delays in HR development after infiltration with *Pst*DC3000(*avrRpt2*) was also observed in mutants defective in SA biosynthesis or signaling [40] (see Figure S3A). We also inoculated the wild type and *lip5-1* mutants with a low dose (OD_600_ = 0.0002) of the avirulent strains and analyzed the growth of the avirulent bacterial pathogens. As shown in Figure S3B, at 5 dpi, the *lip5-1* mutant had ∼10 times higher levels of avirulent bacteria than wild-type plants. Similar increases in bacterial growth upon inoculation of a low dose of the avirulent *Pst*DC3000 strains were also previously observed in Arabidopsis mutants defective in SA biosynthesis or signaling [40].

### The Role of LIP5 in Disease Resistance Is Dependent on Interaction with SKD1

LIP5 has been shown to be a positive regulator of SKD1, a regulator of MVB biogenesis [12]. Both yeast two-hybrid and *in vitro* pull-down assays have shown that Arabidopsis LIP5 is a strong SKD1 interactor and stimulates the SKD1 ATPase activity by 4–5 times [12]. To determine whether the role of LIP5 in plant disease resistance is due to its action as a positive regulator of SKD1, we performed genetic complementation of *lip5-1* mutant with *LIP5* genes. Structural analysis of yeast LIP5 (Vta1) has reveals that the C-terminal domain (CTD) of LIP5 mediates LIP5 dimerization and both subunits are required for interaction with SKD1(VPS4) and for its function as a positive SKD1 regulator [41]. When two conserved CTD residues of yeast Vta1, Tyr-303 and Tyr-310, were mutated into Ala residues, the mutant proteins were deficient in interacting with VPS4 but were normal in maintaining their dimeric structure [41]. The Y303A and Y310A mutant Vta1 protein also failed to stimulate the ATPase activity of VPS4 [41]. The corresponding residues in Arabidopsis LIP5 for Tyr-303 and Tyr-310 of yeast Vta1 are Phe-388 and Phe-395, respectively (see Figure S4). We generated three mutant LIP5 (F388A, F395A and F388A/F395A) in which either or both of the Phe residues were mutated into Ala residues. Using yeast two-hybrid assays, we showed that while wild-type LIP5 is a strong interactor of SKD1, LIP5F388A interacted weakly with SKD1 based on quantitative assays of the *LacZ* reporter gene expression (see Figure S5A). By contrast, no interaction of LIP5F395A or LIP5F388A/F395A with SKD1 was detected in yeast cells using the *LacZ* reporter gene assays (see Figure S5A). As with the corresponding yeast Vtal mutant proteins, dimerization of the Arabidopsis LIP5 mutant proteins was normal (see Figure S5B).

To perform genetic complementation, we placed myc-tagged wild-type and mutant *LIP5* genes into a plant transformation vector under control of the CaMV *35S* promoter and transformed into the *lip5-1* mutant plants. Transformant lines were identified and those expressing similar levels of the *LIP5* transgenes based on western blotting were compared for responses to the virulent *Pst*DC3000 strain. As expected, the *lip5-1* mutant plants were hyper-susceptible to the virulent bacterial pathogen based on the disease symptom development (Figure 2C), chlorophyll contents (see Figure S2B) and bacterial growth (Figure 2D). Transformation of either wild-type *LIP5* or *LIP5F388A* restored disease resistance of *lip5-1* (Figures 2C and 2D). In contrast, transformation of mutant *LIP5F395A* or *LIP5F388A/F395A* failed to restore disease resistance of the *lip5* mutant as indicated from both the severe disease symptoms (Figure 2C) and high bacterial growth (Figure 2D). LIP5F388A, a weak SKD1 interactor (see Figure S5A), also complemented the *lip5* mutant for resistance to the bacterial pathogen (Figures 2C and 2D), most likely due to its overexpression driven by the strong CaMV *35S* promoter in the transgenic plants. These results strongly suggest that interaction with SKD1 is necessary for the critical role of LIP5 in plant resistance to the bacterial pathogen.

### *In Vitro* and *In-Gel* Assays of Phosphorylation of LIP5 by MPK3 and MPK6

**Figure 3:**
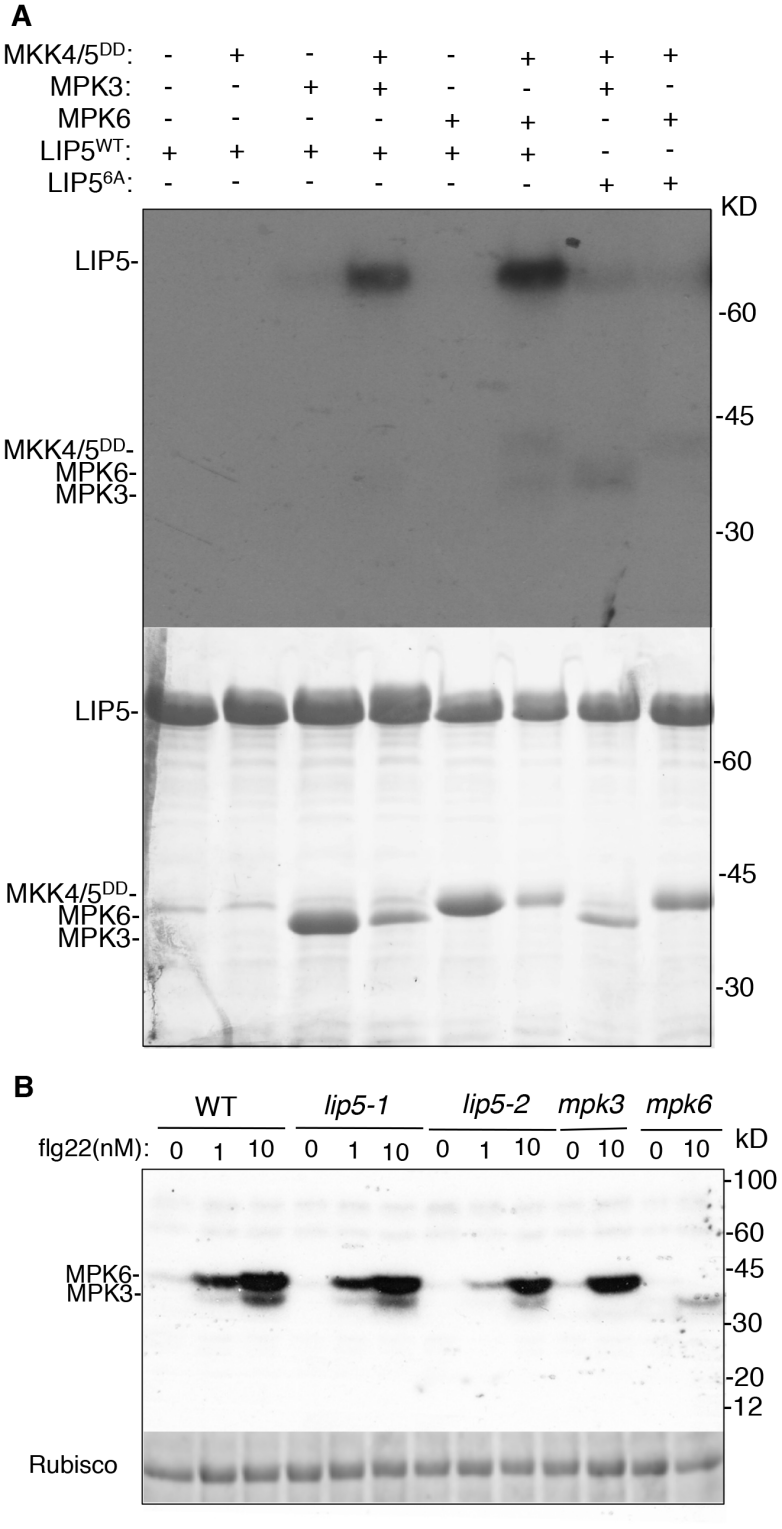
*In vitro* phosphorylation of LIP5 by MPK6 and MPK3. (**A**) Phosphorylation of LIP5 *in vitro* by activated MPK3 and MPK6. Reactions with various proteins omitted (−) were used as controls. LIP5^WT^ but not LIP5^6A^ was phosphorylated in the reactions contain MPK3 or MPK6 in the presence of constitutively active MKK4/5^DD^. Both the radioautograph (upper panel) and Coomassie-stained protein gel (lower panel) are shown. (**B**) Phosphorylation of LIP5 by the native MPK3 and MPK6 extracted from flg22-treated seedlings. Total protein extracts were isolated from wild-type (WT), *lip5*, *mpk3* amd *mpk6* seedlings treated with indicated concentrations of flg22. *In gel* kinase assay was performed using recombinant LIP5 proteins as substrates. Rubisco loading control was from a separate gel loaded with the same amount of proteins as the gel for the in gel kinase assay.

Physical interaction of LIP5 with MPK6 and MPK3 makes LIP5 a possible substrate of the pathogen-responsive MAPKs. Survey of LIP5 protein sequence revealed six proline-directed serine or threonine residues that may act as potential MAPK phosphorylation sites (Ser-73, Thr-153, Ser-254, Ser-285, Ser-307 and Ser-323) (see Figure S4B). To examine phosphorylation, we generated a mutant *LIP5* in which all six proline-directed Ser or Thr residues were mutated into Ala (LIP5^6A^). Yeast two-hybrid assays indicated that mutations of the phosphorylation sites of LIP5 didn't alter its interaction with SKD1 or MPK6 (see Figure S6). We first generated His-tagged LIP5^WT^ and LIP5^6A^ recombinant proteins for *in vitro* phosphorylation assays. As shown in Figure 3A, activated MPK3 and MPK6 both phosphorylated LIP5^WT^. Without co-incubation with the constitutively active MKK4^DD^/MKK5^DD^ for activation, neither of the two MPKs was able to phosphorylate LIP5^WT^ (Figure 3A). By contrast, LIP5^6A^ was not phosphorylated by activated MPK3 or MPK6 (Figure 3A).

We also performed *in-gel* kinase assay to determine phosphorylation of LIP5^WT^ by the native MPKs. In the assay, we embedded recombinant LIP5^WT^ in the SDS-PAGE gel instead of commonly used myelin basic protein. Phosphorylation of embedded LIP5^WT^ was analyzed using total protein extracted from wild type, *lip5*, *mpk3* and *mpk6* mutant seedlings with or without prior treatment with flg22, which activates MPK3 and MPK6. As shown in Figure 3B, a low basal level of phosphorylation of LIP5^WT^ was detected with protein extracts from untreated wild-type seedlings. Greatly Increased phosphorylation of LIP5 by both MPK3 and MPK6 was observed with protein extracts from flg22-treated plants in a concentration-dependent manner (Figure 3B). Similar patterns of phosphorylation of LIP5 were also observed using protein extracts from *lip5* mutants, indicating that the mutants are normal in flg22-induced MPK3 and MPK6 activation. The loss of the kinase bands in the *mpk3* and *mpk6* mutants confirmed the phosphorylation of LIP5 by the respective MPKs in the wild type and *lip5* mutants (Figure 3B). In addition to MPK3 and MPK6, we detected two kinases of approximately 65 and 85 kD that phosphorylates LIP5 at very low levels. Unlike MPK3 and MPK6, the activities of the two unknown kinases were unchanged in flg22-treated plants (Figure 3B).

### *In Vivo* Phosphorylation and Stabilization of LIP5 by Gain-of-Function NtMEK2^DD^ and Pathogen Infection

**Figure 4:**
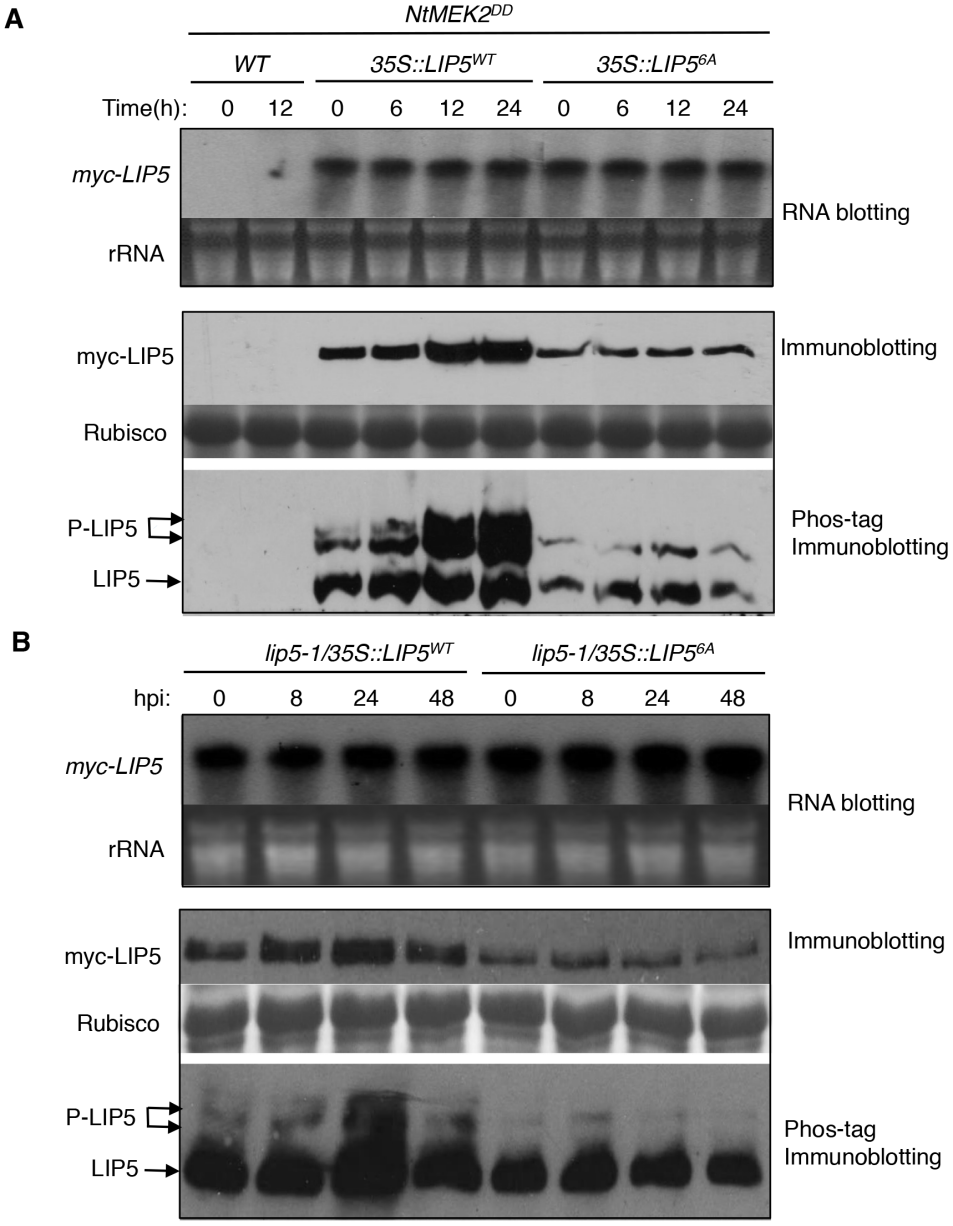
*In vivo* phosphorylation and altered stability of LIP5. (**A**) Phosphorylation and increased stability of LIP5 in gain-of-function *NtMEK2^DD^* plants. Transgenic *NtMEK2^DD^/myc-LIP5^WT^* and *NtMEK2^DD^/myc-LIP5^6A^* lines containing similar levels of *myc-LIP5* transcripts were treated with DEX to induce *NtMEK2^DD^* expression and leaf samples were harvested at indicated time points after DEX treatment. Total RNA was isolated and the levels of *myc-LIP5* transcript were determined by RNA blotting using a ^32^P-labeled *myc* tag DNA fragment as probe (upper). Ethidium bromide staining of rRNA is shown for the assessment of equal loading. Total proteins were also isolated and separated in a regular SDS-PAGE (middle) gel and a Phos-tag SDS-PAGE (lower) gel. After transfer to nitrocellular membranes, myc-LIP5^WT^ and myc-LIP5^6A^ were detected with an anti-myc monoclonal antibody. Rubisco staining of the regular SDS-PAGE gel was used for assessing equal protein loading. (**B**) Phosphorylation and increased stability of LIP5 in pathogen-infected plants. Transgenic *lip5-1/myc-LIP5^WT^* and *lip5-1/myc-LIP5^6A^* lines containing similar levels of *myc-LIP5* transcripts were inoculated with *Pst*DC3000 (OD_600_ = 0.02 in 10 mM MgCl_2_). Samples were collected at indicated hours post-inoculation (hpi) for total RNA and protein isolation. RNA blotting, regulator immunoblotting and Phos-tag immunoblotting were performed as in **A**.

To determine *in vivo* phosphorylation of LIP5 by MPK3 and MPK6, we subcloned *myc*-tagged *LIP5^WT^* and *LIP5^6A^* into plant transformation vector under control of the CaMV *35S* promoter and transformed into wild-type plants. These transgenic *myc-LIP5^WT^* and *myc-LIP5^6A^* lines were then crossed with the dexamethasone (DEX)-inducible promoter-driven gain-of-function *NtMEK2^DD^* (*GVG*-Nt*MEK2^DD^*), which, like Arabidopsis AtMKK4^DD^ and AtMKK5^DD^, can activate MPK3 and MPK6 through phosphorylation [42], [43], [44]. Transgenic *myc-LIP5/NtMEK2^DD^* lines containing similar levels of *LIP5* transgene transcripts were identified by RNA blotting using *myc*-tag DNA as probe (Figure 4). Total proteins were also isolated from these transgenic plants and analyzed by western blotting using anti-myc antibody after separation on a SDS-PAGE gel. As shown in Figure 4A, even with similar transcript levels, the protein levels of myc-LIP5^WT^ were higher than those of myc-LIP5^6A^ even before DEX induction of *NtMEK2^DD^*. After DEX treatment, the protein levels of myc-LIP5^WT^ were further increased while those of myc-LIP5^6A^ remained unchanged (Figure 4A). By 24 hours after DEX treatment, the protein level of myc-LIP5^WT^ was at least 5–6 times higher than that of myc-LIP5^6A^ (Figure 4A). These results suggest that the protein stability of LIP5 is positively regulated by the basal activities of MAPKs and is further enhanced by increased MAPK activation by gain-of-function NtMEK2^DD^.

To provide direct evidence that LIP5 is phosphorylated *in vivo*, we performed the Phos-tag mobility shift assay, which is based on the reduced mobility of phospho-proteins due to their binding to the Phos-tag reagents in the SDS-PAGE gel matrix [45]. Detached leaves were treated with DEX and protein samples collected at different time points were separated on Phos-tag Acrylamide and myc-LIP5 proteins were detected by western blotting using anti-myc antibody. Without DEX treatment, we detected two major bands of LIP5^WT^ and a minor band that was most retarded on the gel (Figure 4A). After DEX treatment, the intensities of the two retarded bands increased greatly, although the level of the least retarded band also increased significantly (Figure 4A). By contrast, we detected two bands for LIP5^6A^ and their intensities didn't change significantly after DEX treatment (Figure 4A). Based on these results, the least retarded band is mostly likely the unphosphorylated LIP5 while the two retarded bands are phosphorylated LIP5. Since even the LIP5^6A^ had a retarded band, there appears to be other sites in LIP5 besides the 6 proline-directed Ser and Thr that are subjected to phosphorylation by unknown kinases. Phosphorylation of proline-directed Ser/Thr residues by activated MAPKs caused further phosphorylation of LIP5^WT^, leading to further reduction in mobility on the Phos-tag gel (Figure 4A). Furthermore, increased phosphorylation of LIP5^WT^ after DEX treatment was accompanied by a substantial increase in the protein level as indicated by the combined intensities of the three detected protein bands (Figure 4A). No such increase in protein levels was observed in DEX-treated plants expressing the mutant LIP5^6A^ protein (Figure 4A).

To determine whether LIP5 was also phosphorylated *in vivo* after pathogen infection, we inoculated with *Pst*DC3000 transgenic *myc-LIP5^WT^* and *myc-LIP5^6A^* lines expressing similar levels of the respective *myc-LIP5* transgenes based on RNA blotting (Figure 4B). Total proteins were again isolated from these transgenic plants and analyzed by western blotting using anti-myc antibody after separation on a SDS-PAGE gel. As shown in Figure 4B, despite similar transcript levels, the protein level of myc-LIP5^WT^ was substantially higher than that of myc-LIP5^6A^ even before pathogen infection. After *Pst*DC3000 inoculation, the protein levels of myc-LIP5^WT^ were further increased while those of myc-LIP5^6A^ were unchanged (Figure 4B). At 24 hpi, the protein level of myc-LIP5^WT^ was at least 4–5 times higher than that of myc-LIP5^6A^ (Figure 4B). Furthermore, the Phos-tag mobility shift assay revealed increased phosphorylation of LIP5^WT^ after *Pst*DC3000, with the highest levels at 24 hpi (Figure 4B). No such increase in phosphorylation of LIP5^6A^ was observed after *Pst*DC3000 (Figure 4B). These results indicate that the protein stability of LIP5 is positively regulated by its basal level of phosphorylation and can be further enhanced by increased phosphorylation after pathogen infection.

To confirm that retarded LIP5 protein bands on the Phos-tag gels were resulted from phosphorylation, we treated protein extracts from DEX-treated or pathogen-infected transgenic myc-LIP5^WT^ plants with calf intestinal phosphatase (CIP) prior to electrophoresis. The treatment of CIP led to almost complete collapse of the retarded bands and the collapse could be blocked by inclusion of a protein phosphorylation inhibitor (see Figure S7). This result indicates that retarded migration of LIP5^WT^ is due to phosphorylation.

### Phosphorylation-Regulated LIP5 Protein Stability Is Critical for Plant Basal Resistance

**Figure 5:**
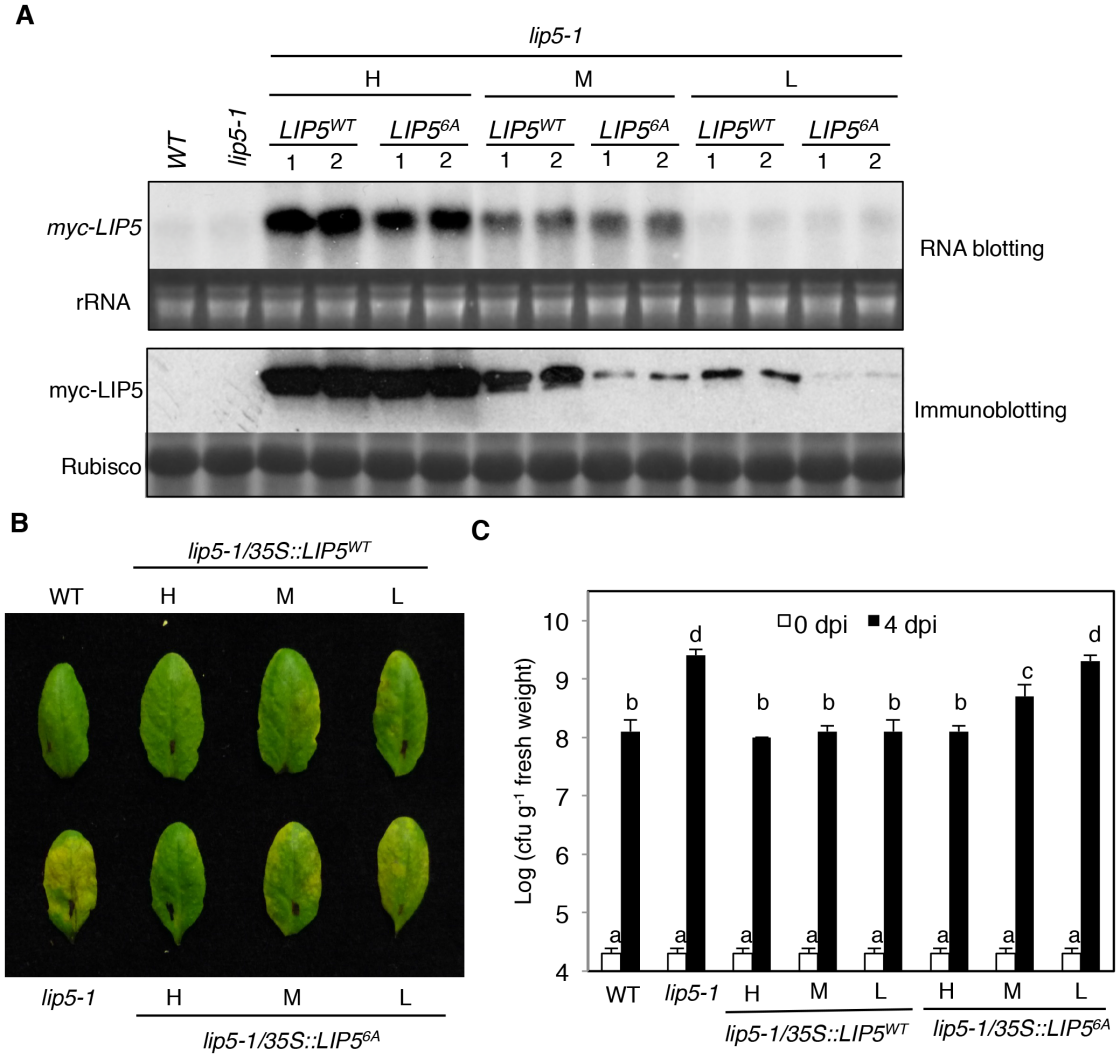
Phosphorylation-regulated LIP5 protein stability and plant basal resistance. (**A**) Comparison of *myc-LIP5* transcript and myc-LIP5 protein levels in *lip5-1/myc-LIP5^WT^* and *lip5-1/myc-LIP5^6A^* lines. Two independent *lip5-1/myc-LIP5^WT^* and *lip5-1/myc-LIP5^6A^* lines each with similarly high (H), medium (M) and low (L) levels of *myc-LIP5* transcripts were identified by RNA blotting using a ^32^P-labeled *myc* tag DNA fragment as probe. Ethidium bromide staining of rRNA was shown for the assessment of equal loading. Total proteins were also isolated from the same lines and the myc-LIP5 protein levels were detected by regular immunoblotting with an anti-myc monoclonal antibody. Rubisco staining was also included for assessing equal protein loading. Untransformed wild type (WT) and *lip5-1* mutant were included as negative controls. (**B**) Disease symptom development after *Pst*DC3000 inoculation. WT, *lip5-1* and *lip5-1/myc-LIP5^WT^* and *lip5-1/myc-LIP5^6A^* lines with similarly high (H), medium (M) and low (L) levels of *myc-LIP5* transcripts were inoculated with *Pst*DC3000 (OD_600_ = 0.0002 in 10 mM MgCl_2_). Pictures of representative leaves were taken at 4 dpi. (**C**) Bacterial growth. Pathogen inoculation of wild-type and mutant plants was performed as in B. Samples were taken at 0 or 4 dpi to determine the bacterial growth. The means and standard errors were calculated from 10 plants for each mutant. According to Duncan's multiple range test (P = 0.05), means of colony-forming units (cfu) do not differ if they are indicated with the same letter.

To determine the importance of LIP5 phosphorylation by the pathogen-responsive MPKs, we compared the ability of myc-LIP5^WT^ and myc-LIP5^6A^ in complementing the *lip5* mutant phenotype. We initially used the 1.5 kb upstream promoter sequence of *LIP5* for driving the *myc-LIP5^WT^* and *myc-LIP5^6A^* transgene. Surprisingly, unlike the constitutive CaMV *35S* promoter-driven *myc-LIP5^WT^* construct, the *LIP5* promoter-driven *myc-LIP5^WT^* construct (*P_LIP5_::myc-LIP5^WT^*) failed to complement the *lip5* mutant phenotype. RNA blotting using *LIP5* as probe failed to detect the *LIP5* transcripts and western blotting detected no accumulation of myc-LIP5^WT^ in the *lip5-1/P_LIP5_::myc-LIP5^WT^* lines (data not shown). This result indicated that additional non-coding sequences other than the 1.5 kb upstream sequence are necessary for sufficient levels of *LIP5* expression. For this reason, we again used the CaMV *35S*-driven *LIP5* (*35S::LIP5^WT^* and *35S::LIP5^6A^*) constructs and obtained more than 30 independent lines for each construct in the *lip5-1* mutant background. Using RNA blotting with the *myc* tag DNA as probe, we identified three types of transgenic lines for each construct that contained high (H), medium (M) and low (L) levels of the *myc-LIP5* transgene (Figure 5A). Protein immunoblotting using an anti-myc antibody revealed that there were similarly high levels of myc-LIP5 proteins in the transgenic *lip5-1/35S::LIP5^WT^-H* and *lip5-1/35S::LIP5^6A^-H* lines, which contained relatively high levels of *myc-LIP5* transcripts (Figure 5A). When these *lip5* transgenic lines were inoculated with *Pst*DC3000, the resistance was fully restored to the levels of wild-type plants based on both disease symptom development and bacterial growth (Figures 5B and 5C). On the other hand, when comparing the transgenic lines that contained medium levels of *myc-LIP5* transcripts, we observed that the protein levels of myc-LIP5^WT^ in the *lip5-1/35S::LIP5^WT^-M* lines were substantially higher than those of myc-LIP5^6A^ in the *lip5-1/35S::LIP5^6A^-M* lines (Figure 5A). Inoculation with *Pst*DC3000 further showed that while the disease resistance was fully restored in the *lip5-1/35S::LIP5^WT^-M* lines, it was only partially restored in the *lip5-1/35S::LIP5^6A^-M* lines (Figure 5B and 5C). Finally, when comparing the transgenic lines that contained low levels of *myc-LIP5* transcripts, we again observed that the protein levels of myc-LIP5^WT^ in the *lip5-1/35S::LIP5^WT^-L* lines were substantially higher than those of LIP5^6A^ in the *lip5-1/35S::LIP5^6A^-L* lines (Figure 5A). Furthermore, while the disease resistance was restored in the *lip5-1/35S::LIP5^WT^-L* lines, it was not restored at all in the *lip5-1/35S::LIP5^6A^-L* lines (Figure 5B and 5C). Thus, when the LIP5 transgene was expressed at medium or low levels, its enhanced stability by phosphorylation became increasingly important for its full ability to complement the *lip5* mutant phenotype. It should be noted that based on RNA blotting using *LIP5* DNA fragment as probe, the transcript levels of the native *LIP5* gene in pathogen-infected wild-type plants were even lower than those of *myc-LIP5* in the *lip5-1/35S::LIP5^WT^-L* and *lip5-1/35S::LIP5^6A^-L* lines (see Figure S8). Therefore, the results from the comparative analysis of the transgenic *lip5-1/35S::LIP5^WT^-L* and *lip5-1/35S::LIP5^6A^-L* lines for the role of phosphorylation-regulated LIP5 stability is applicable to the native LIP5 protein in wild-type plants.

### Phenotypes of *lip5* Mutants in PTI and SA-Mediated Defense

**Figure 6:**
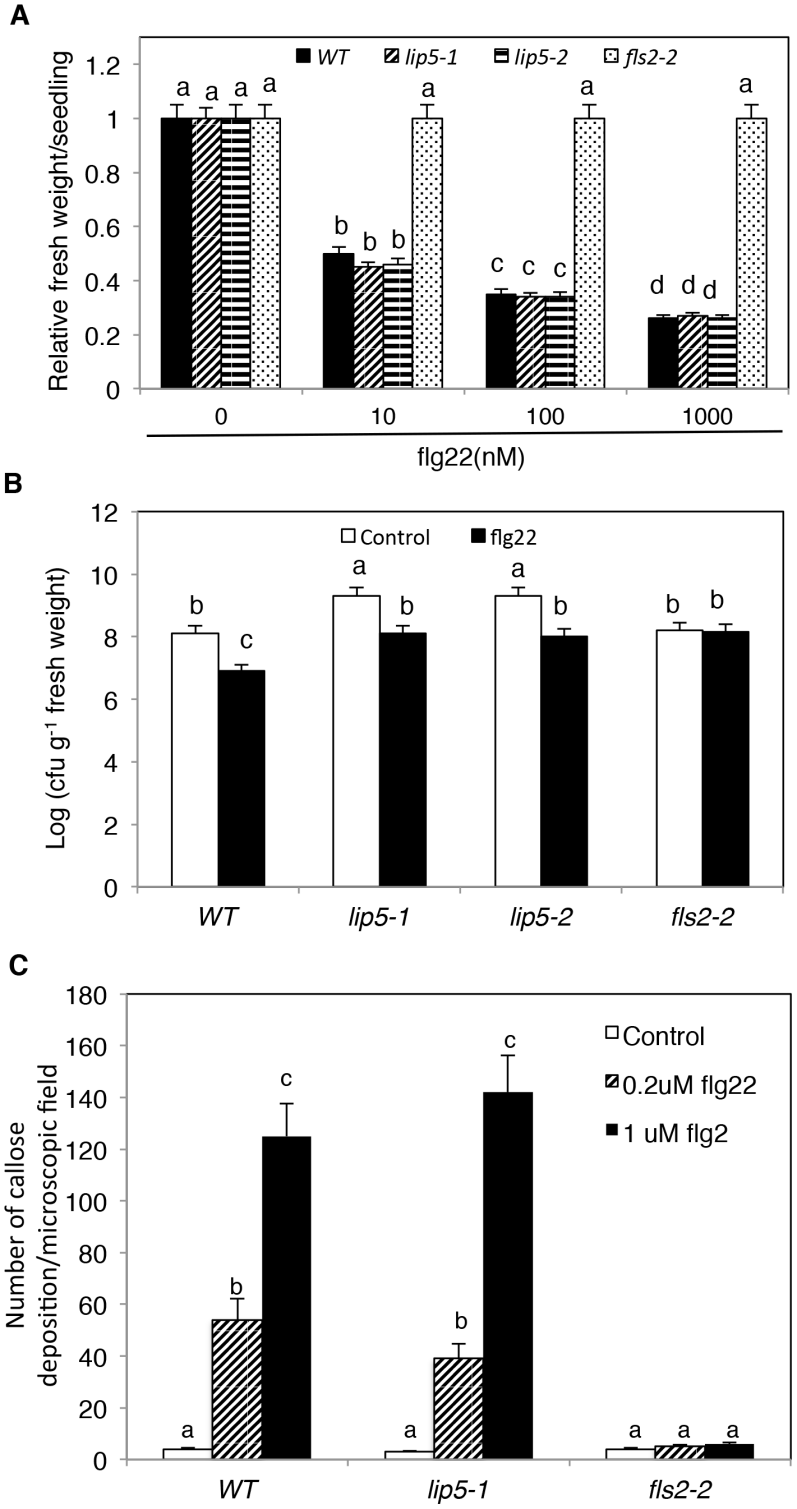
Responses to flg22 by the *lip5* and *fls2* mutants. (**A**) Growth inhibition by flg22. Five-days old wild-type (WT), *lip5-1*, *lip5-2* and *fls2-2* mutant seedlings were transferred to ½ MS liquid medium containing indicated concentrations of flg22, in 12-well growing plates. Seedlings were further grown for 7 days before fresh weight were measured. (**B**) Induction of disease resistance by flg22. Leaves of 5 weeks old WT, *lip5* and *fls2* mutant plants were preinfiltrated with 1 µM flg22. Uninfiltrated leaves were used as control. One day after the treatment, the plants were infiltrated with a suspension of *Pst*DC3000 (OD_600_ = 0.0002 in 10 mM MgCl_2_). Samples were taken at 4 dpi to determine the bacterial growth. The means and standard errors were calculated from 10 plants. According to Duncan's multiple range test (P = 0.05), means of colony-forming units (cfu) do not differ if they are indicated with the same letter. (**C**) Induction of Callose Deposition by flg22. Ten-days-old wild type (WT), *lip5-1* and *fls2-2* mutant seedlings were transferred to ½ MS liquid medium containing 0 (control), 0.2 or 1 µM flg22. After 24 hours, seedlings were stained by aniline blue and callose deposition was observed under a Nikon eclipse E800 epifluorescence microscopy and the numbers were manually quantified.

To study how *lip5* mutants are compromised in disease resistance, we compared the mutants with wild type for PTI and SA-mediated defense, both of which are critical for resistance to the bacterial pathogen [46], [47]. To test whether *lip5* mutants had normal onset of PTI, we employed flg22, a 22-amino-acid PTI peptide elicitor from bacterial flagellin. Application of flg22 induces transcriptional and translational reprogramming and cellular responses that prime the defense pathways in Arabidopsis [48]. Pre-application of flg22 can substantially decrease the growth of *P. syringae* in *Arabidopsis* leaves, and prolonged treatment of flg22 of *Arabidopsis* seedlings results in growth inhibition [49]. As shown in Figure 6A, prolonged incubation in flg22 led to inhibition of seedling growth of wild-type and *lip5* mutants in a concentration-dependent manner. By contrast, no inhibition of seedling growth by flg22 was observed in the *fls2-2* mutant for the Arabidopsis receptor required for perception of the bacterial flagellin elicitor [50] (Figures 6A). Likewise, pre-application of flg22 reduced the growth of the virulent bacterial pathogen by ∼15 fold in both the wild type and *lip5* mutants but not in the *fls2-2* mutant (Figure 6B). PTI is also associated with increased callose deposition [51]. To test whether *lip5* mutant plants were normal in flg22-induced callose deposition, we treated wild-type, *lip5-1* and *fls2-2* mutant seedlings with 0, 0.2 and 1 µM flg22 and compared the numbers of callose deposition. As shown in Figure 6C, both wild-type and *lip5-1* mutant seedlings showed similar concentration-dependent increases in callose deposition following flg22 treatment. By contrast, no flg22-induced increase in callose deposition was observed in the *fls2-2* mutant (Figure 6C). Induction of a number of early PTI WRKY marker genes and *PATHOGENESIS-RELATED PROTEIN* (*PR*) late marker genes by flg22 was also largely normal in the *lip5* mutants (see Figure S9). These results indicated that *lip5* mutants were normal in PTI.

**Figure 7:**
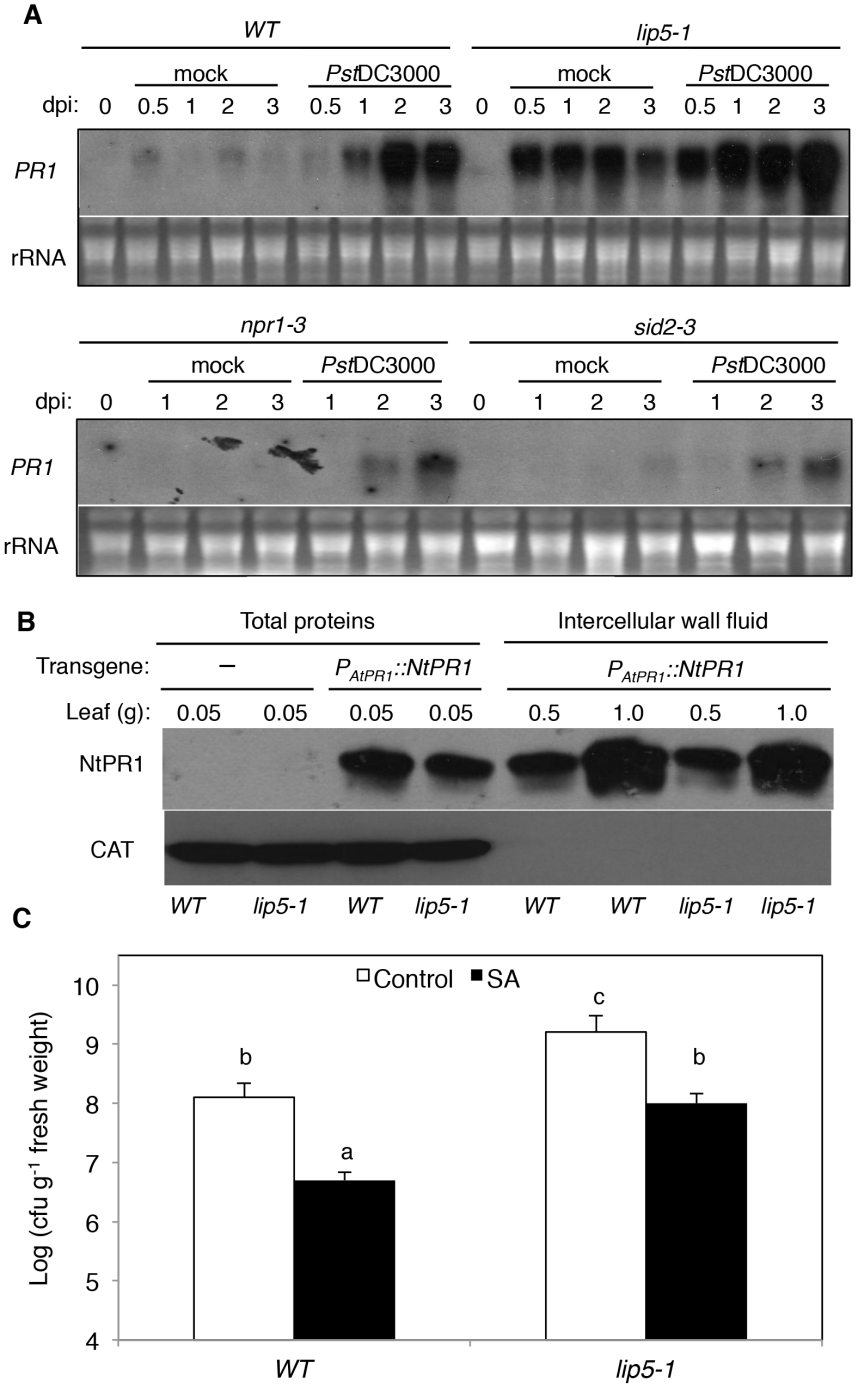
SA-mediated defense responses. (**A**) Pathogen-induced *PR1* gene expression. Five-weeks old wild type (WT), *lip5-1*, *npr1-3* and *sid2-3* mutant plants were infiltrated with 10 mM MgCl_2_ (mock) or *Pst*DC3000 (OD_600_ = 0.0002 in 10 mM MgCl_2_). Samples were collected at indicated days post-inoculation (dpi) for total RNA isolation and RNA blotting analysis of *PR1* gene expression with a ^32^P-labeled *PR1* probe. Ethidium bromide staining of rRNA is shown for the assessment of equal loading. (**B**) PR1 protein secretion. Total proteins or intercellular wall fluid (IWF) were isolated 24 hours after spraying 1 mM SA from transgenic wild type (WT) and *lip5-1* mutant plants harboring the *P_AtPR1_::NtPR1* construct and analyzed by western blotting using the 33G1 monoclonal antibody that specifically recognizes NtPR1. The total protein and IWF were also analyzed by western blotting using an anti-catalase (CAT) monoclonal antibody for examining possible contamination of IWF by intracellular proteins. (**C**) SA-induced disease resistance. Wild type (WT) and *lip5-1* mutant plants were sprayed with H_2_O (control) or 1 mM SA (SA). One day after treatment, the plants were infiltrated with *Pst*DC3000 (OD_600_ = 0.0002 in 10 mM MgCl_2_) and bacteria growth was determined at 4 dpi. The means and standard errors were calculated from 10 plants. According to Duncan's multiple range test (P = 0.05), means of colony-forming units (cfu) do not differ if they are indicated with the same letter.

SA-mediated defense plays an important role in Arabidopsis resistance to *P. syringae* [52], [53], [54]. SA-mediated defense is associated with induction of *PR* genes, which are observed in both SA-treated and *Pst*DC3000-infected plants [52]. The *sid2* and *npr1* mutants defective in SA biosynthesis and signaling, respectively, are compromised in pathogen-induced *PR* gene expression [38], [39]. To test if SA pathway is compromised in *lip5* mutants, we analyzed induction of SA-regulated *PR1* expression after infiltration with 10 mM MgCl_2_ (mock inoculation) or with virulent *Pst*DC3000. No *PR1* transcript was detected in either healthy wild-type or *lip5-1* mutant plants (Figure 7A). In wild-type plants, little *PR1* transcripts were detected after mock inoculation but were induced after inoculation with the bacterial pathogen (Figure 7A). In the *lip5-1* mutant plants, intriguingly, *PR1* transcripts were elevated substantially even in mock-inoculated leaves (Figure 7A). Elevated *PR1* transcripts in mock-inoculated *lip5-1* mutant was likely caused by infiltration-caused wounding as spraying or soaking of the leaves of the same mutant led to only slight induction of *PR1* (see Figure S9). Furthermore, induction of *PR1* was faster and to significantly higher levels in the *lip5-1* mutant plants than in wild-type plants following *Pst*DC3000 inoculation (Figure 7A). By contrast, pathogen-induced *PR1* expression was greatly reduced in both *sid2-3* and *npr1-3* mutants (Figure 7A). To determine whether pathogen-induced PR1 proteins are normally secreted in the *lip5* mutant plants, we generated a construct in which the tobacco acidic *NtPR1* gene is under control of the Arabidopsis *PR1* gene promoter. The construct was then transformed into both wild type and *lip5-1* mutant. Pathogen-induced production of tobacco PR1 proteins and their accumulation in intercellular wash fluid were determined by western blotting using a monoclonal antibody (33G1) recognizing tobacco PR1 (Chen et al., 1993). As shown in Figure 7B, secretion of transgenic tobacco PR1 protein was normal in *lip5-1* mutant, as shown in western blot of intercellular wash fluid after 1 mM SA treatment (Figure 7B). These results indicated that the *lip5* mutant plants are competent in SA-mediated *PR1* expression and PR1 protein secretion.

To further determine the responsiveness of the *lip5* mutant plants to SA, we compared them with wild-type plants for SA-induced disease resistance. In wild-type plants, pretreatment of 1 mM SA reduced the growth of the virulent *Pst*DC3000 strain by ∼15-fold (Figure 7C). Similarly reduced growth of the bacterial pathogen was observed in the *lip5-1* mutant plants after SA treatment (Figure 7C). These results support that the *lip5* mutants are competent in SA-mediated defense.

### Compromised Phenotype of *lip5* in Pathogen-Induced Vesicle Trafficking

**Figure 8:**
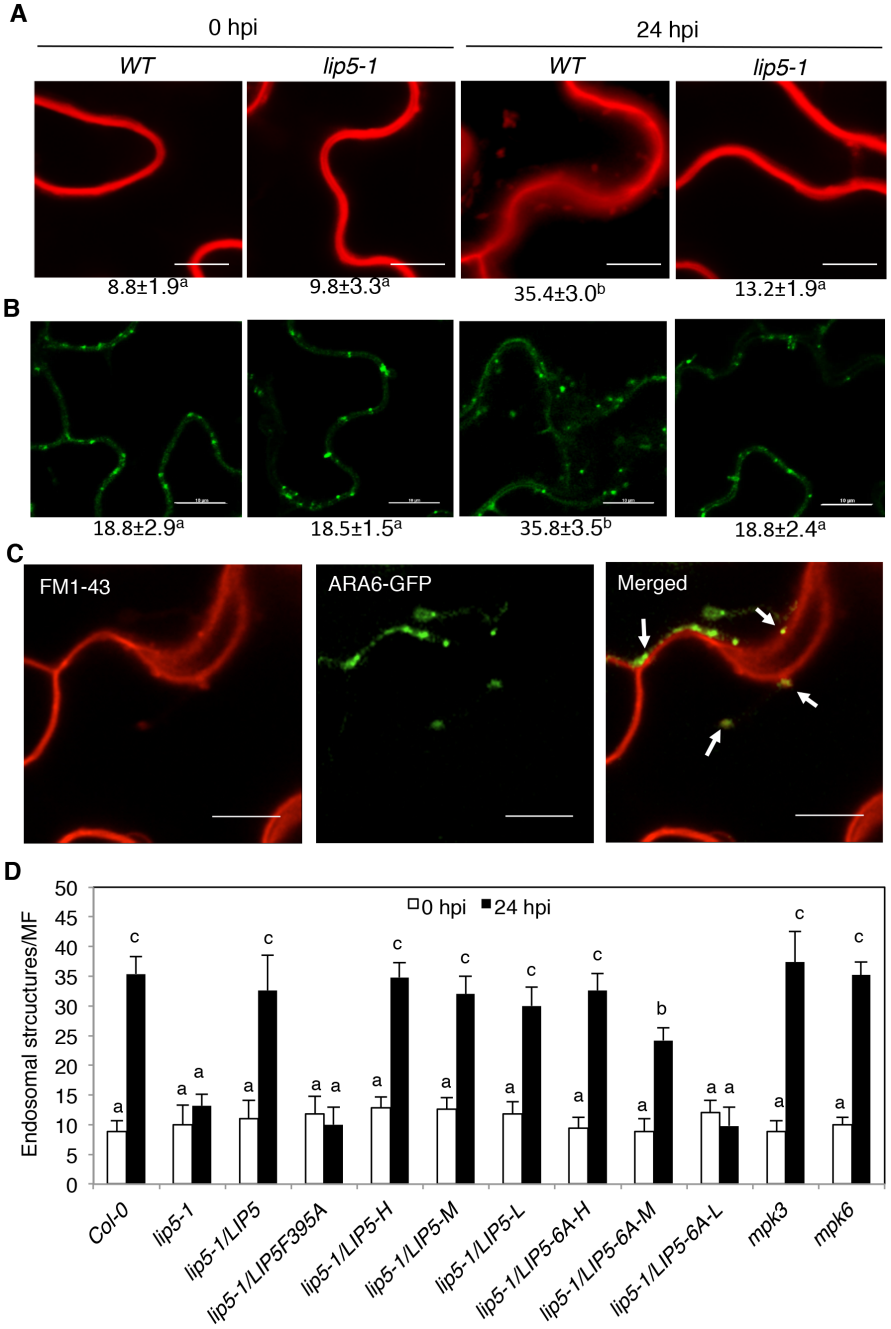
Pathogen-induced vesicle trafficking. (**A**) Confocal sections of Arabidopsis leaf cells labeled with FM1–43. Leaves of wild-type (WT), *lip5-1* and *lip5-2* mutant were collected at 0 and 24 hours post inoculation (hpi) of *Pst*DC3000 and were stained in 20 µM FM1–43 for 1 hour in room temperature, washed and observed under a Zeiss LSM710 confocal microscope. Bars = 10 µm. (**B**) Confocal sections of leaf cells of transgenic *ARA6-GFP* Arabidopsis. Leaves of transgenic WT and *lip5-1* plants expressing *ARA6-GFP* were collected at 0 and 24 hpi of *Pst*DC3000 for observation of ARA6-GFP fluorescent signals under a Zeiss LSM710 confocal microscope. Bars = 10 µm. The numbers of internalized fluorescent endosomal structures per microscopic field (MF, 60 µm×60 µm) were indicated below the images in A and B. Means and SE were calculated from three experiments. According to Duncan's multiple range test (*P* = 0.05), means do not differ significantly if they are indicated with the same letter. (**C**) Colocalization of FM1–43 labeled fluorescent signals with ARA6-GFP. Leaves of transgenic WT plants expressing ARA6-GFP were collected at 24 hpi of *Pst*DC3000 and were stained in FM1–43 before observation of the FM1–43 and ARA6-GFP fluorescent signals. About 40% of the FM1–43–positive compartments were also labeled by ARA6-GFP (arrows). Bars = 10 µm. (**D**) Numbers of internalized fluorescent endosomal structures. Pathogen inoculation and FM1–43 staining of WT, mutants and transformants were performed as in **A**. The numbers of internalized fluorescent endosomal structures per MF (60 µm×60 µm) were determined under a confocal microscope. The means and standard errors were calculated from at least 20 MFs of three independent samples. According to Duncan's multiple range test (P = 0.05), means of colony-forming units (cfu) do not differ if they are indicated with the same letter.

As a positive regulator of SKD1 in MVB biogenesis, the critical role of LIP5 in plant immune system is likely through regulation of cellular vesicle trafficking during plant-pathogen interactions. To test this, we compared wild-type and *lip5* mutant plants for pathogen-induced endocytosis using the styryl dye FM1–43 as a fluorescent endocytosis marker. The membrane-selective FM1–43, which fluoresces significantly only in a lipid-rich membrane, is unable to cross membrane because of the amphiphilic nature but can enter the cells by endocytic vesicles invaginated from the plasma membrane [55], [56]. In uninfected wild-type plants, FM1–43 strongly labeled the plasma membrane as a result of the association of the dye with the lipid phase (Figure 8A). After *Pst*DC3000 inoculation, the sharp fluorescence at the plasma membrane was reduced and became diffusive (Figure 8A). In addition, there was approximately 4-fold increase in the number of internalized fluorescent vesicles in pathogen-infected wild-type plants when compared to uninfected plants (Figure 8A). In the *lip5-1* mutant plants, strong labeling of plasma membrane and low numbers of internalized fluorescent vesicles were also observed as in wild-type plants (Figure 8A). Unlike in wild-type plants, however, pathogen-induced reduction of fluorescence at the plasma membrane and increase in internalized fluorescent vesicles were almost completely abolished in the *lip5-1* mutant plants (Figures 8A). These results indicate that pathogen infection stimulates endocytosis and vesicle trafficking in host cells in a LIP5-dependent manner.

To further analyze the role of LIP5 in pathogen-induced MVB formation, we generated transgenic wild-type and *lip5-1* mutant plants expressing a GFP-fused MVB marker protein, the ARA6/RabF1 GTPase [12]. The transgenic plants were inoculated with the virulent *Pst*DC3000 bacterial pathogens and observed for the ARA6-GFP signals at 0 and 24 hpi. At 0 hpi, we detected similar levels of punctate ARA6-GFP signals in the wild type and *lip5* mutant plants (Figure 8). After *Pst*DC3000 inoculation, the numbers of punctate ARA6-GFP signals were doubled in wild-type plants but were little changed in the *lip5* mutant plants (Figure 8B). These results support that pathogen infection induces MVB biogenesis in plant cells in a LIP5-dependent manner. Interestingly, the numbers of ARA6-GFP-labeled vesicles were substantially higher than those of internalized FM1–43-stained vesicles at 0 hpi and, as a result of the higher basal levels, the induction of ARA6-GFP-labeled vesicles was smaller than that of internalized FM1–43-stained vesicles after *Pst*DC3000 infection (Figure 8A & 8B). This discrepancy might result from the fact that FM1–43 stained mostly endocytic vesicles invaginated from the plasma membrane, which are apparently highly responsive to pathogen infection, while constitutively expressed ARA6-GFP would label not only MVBs derived from endocytic vesicles but also MVBs derived from constitutive vesicle trafficking pathways invaginated from other subcellular compartments.

To determine whether internalized FM1–43 fluorescent vesicles, which increased in wild-type plants after *Pst*DC3000 infection, are related to MVBs, we analyzed co-localization of the vesicles with those labeled by ARA6-GFP. Transgenic plants expressing the ARA6-GFP MVB marker were inoculated with *Pst*DC3000 and analyzed for increased vesicle trafficking using FM1–43. As shown in Figure 8C, internalized FM1–43 fluorescent vesicles were observed in pathogen-infected plants and approximately 40% of these FM1–43 punctate spots were also labeled with ARA6-GFP.

To analyze the role of LIP5 interaction with SKD1 and LIP5 phosphorylation in pathogen-induced vesicle trafficking, we examined internalized FM1–43 fluorescent vesicles in the *lip5-1* mutant complemented with the *LIP5F395A* or *LIP5^6A^* mutant gene. As shown in Figure 8D, will-type LIP5 at various levels could fully complement the *lip5* mutant for pathogen-induced formation of FM1–43-stained vesicle structures. Likewise, mutant LIP5^6A^ at high levels (LIP5-6A-H) could fully restore the vesicle numbers in pathogen-inoculated *lip5* mutant (Figure 8D). Mutant LIP56A at medium levels (LIP5-6A-M), on the other hand, could only partially restore the vesicle numbers in pathogen-inoculated *lip5* mutant (Figure 8D). By contrast, mutant LIP5F395A even at high levels and mutant LIP56A at low levels (LIP5-6A-L) were ineffective in restoring the vesicle numbers in pathogen-inoculated *lip5* mutant (Figure 8D). These results indicate that both the interaction with SKD1 and phosphorylation-dependent stability of LIP5 are critical for pathogen-induced vesicle trafficking. We also found that both the *mks3* and *mks6* mutants were normal in pathogen-induced formation of FM1–43-stained vesicles (Figure 8D). It is most likely that lack of compromised phenotypes in pathogen-induced vesicle trafficking in the *mpk3* and *mpk6* mutants was due to the functional redundancy of the two kinases as previously observed [30], [57], [58].

**Figure 9:**
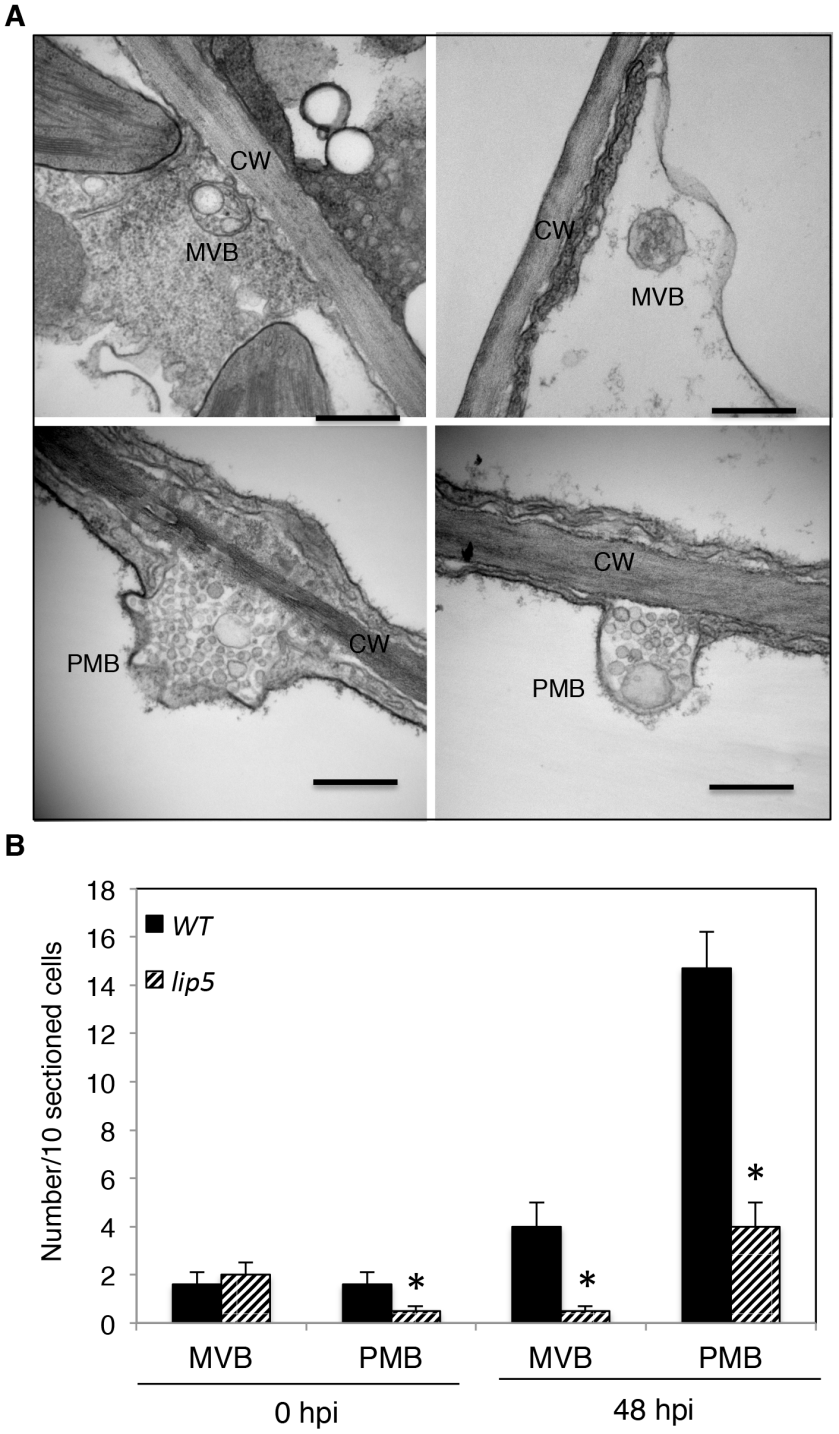
Pathogen-induced MVB and PMB formation. (**A**) TEM sections of Arabidopsis leaf cells after *Pst*DC3000 infection. Both MVBs in cytosol (upper left) or in vacuole (upper right) and PMBs (lower) were observed in wild-type leaf cells collected at 48 hpi of *Pst*DC3000. CW, cell wall; Bars = 0.2 µm. (**B**) Numbers of MVBs and PMBs per 10 sectioned cells. The means and standard errors were from three independent leaf samples. The asterisks indicate numbers of MVBs or PMBs in the *lip5* mutant that are significantly different from corresponding numbers in wild type (*t* test, P = 0.05).

A number of reported studies have implicated MVBs in a cell wall-associated defense responses in barley leaves to the pathogenic powdery mildew fungus through fusion with the plasma membrane to release internal vesicles into the paramural space. To further analyze pathogen-induced, LIP5-regulated MVB trafficking, we employed transmission electron microscopy (TEM) for detection and ultrastructural characterization of MVBs or related vesicle bodies in *Pst*DC3000-infected Arabidopsis plants. As shown in Figure 9, TEM revealed occurrence of spherical MVBs containing small vesicles in the lumen in both the cytosol and vacuole. The TEM study also revealed exosome-like paramural vesicles situated between the plasma membrane and the cell wall, similar to the paramural bodies (PMVs) observed in barley leaves attacked by the pathogenic powdery mildew fungus (Figure 9). To quantify the effects of *Pst*DC3000 infection and disruption of LIP5 on the occurrence of the vesicular bodies, we estimated the average numbers of MVBs and PMBs per 10 sectioned cells in both wild plants and *lip5-1* mutants at both 0 and 48 hpi. At 0 hpi, both wild-type and *lip5* mutant plants had 1–2 MVBs or PMBs per 10-sectioned cells. At 48 hpi, the numbers of MVBs and PMBs per 10-sectioned cells in wild-type plants increased to 4 and 14, respectively (Figure 9). In contrast, the numbers of MVBs and PMBs per 10-sectioned cells in the *lip5* mutant plants were only 1 and 3, respectively (Figure 9). Thus, PstDC3000 infection increased the number of MVBs and to a greater extent, PMVs and this pathogen-induced MVB/PMB formation was largely LIP5-dependent.

### Expression and Subcellular Localization of LIP5

We monitored the expression level of *LIP5* and its activating target *SKD1* during *Pst*DC3000 infection using RNA blotting. In wide-type plants, the levels of *LIP5* transcript were slightly and transiently increased after mock inoculation (see Figure S10). In *Pst*DC3000-inoculated plants, the increase in the levels of *LIP5* transcript was even stronger, particularly at 2 and 3 dpi (see Figure S10). The levels of *SKD1* transcripts was unchanged in mock-inoculated plants but also significantly elevated in *Pst*DC3000-infected plants (see Figure S10). Thus, expression of both *LIP5* and *SKD1* was responsive to pathogen infection.

**Figure 10:**
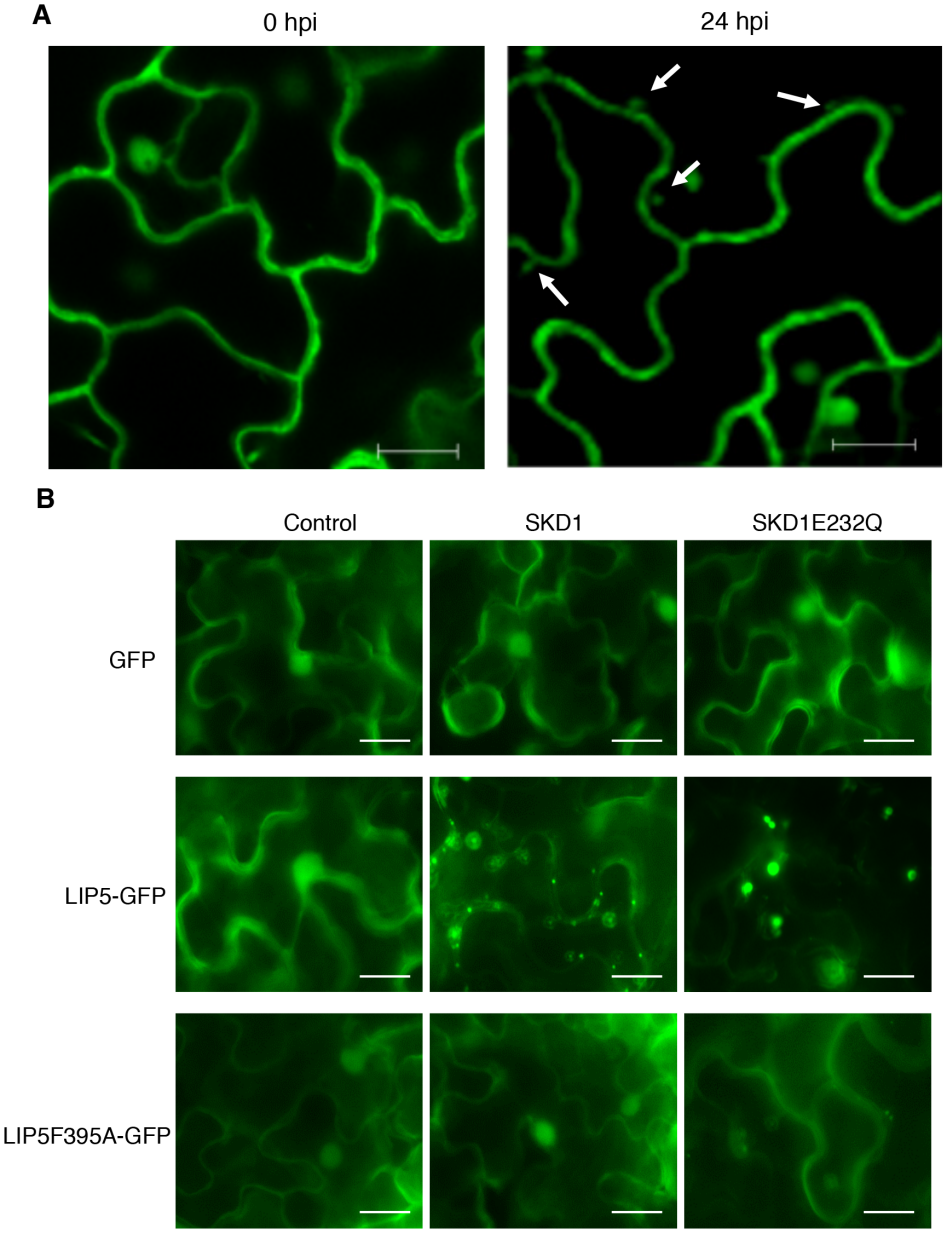
Subcellular localization of LIP5. (**A**) Effect of pathogen infection on LIP5 subcellular localization. Transgenic Arabidopsis plants expressing *LIP5-GFP* was infiltrated with *Pst*DC3000 (OD_600_ = 0.0002 in 10 mM MgCl_2_). Leaf samples were collected at indicated hours post inoculation (dpi) of *Pst*DC3000 and observed under a confocal microscope. Internal fluorescent vesicle structures observed at 24 hpi are indicated by arrows. Bars = 10 µm. (**B**) Effect of SKD1 on subcellular localization of LIP5. Expression constructs of LIP5-GFP or LIP5F395A were coexpressed transiently in *N. benthamiana* with *SKD1* or *SKD1E232Q* under control of a dexamethasone (DEX)-inducible promoter. Two days after *Agrobacterium* infiltration, the tobacco leaves were treated with 30 µM DEX to induce SKD1 expression and LIP5-GFP subcellular localization was examined under a fluorescence microscope 24 hours later. Bars = 20 µm.

To examine the subcellular localization of LIP5, we generated a *LIP5-GFP* fusion gene and transformed into the *lip5-1* mutant. Western blotting using an anti-GFP monoclonal antibody detected a protein band in the transgenic plants but not in untransformed plants with a molecular weight expected for that of LIP5-GFP (see Figure S11A), suggesting that the fusion protein was intact. Transformation of the *LIP5-GFP* gene completely restored the disease resistance of *lip5-1* (see Figure S11B & S11C), indicating the fusion protein is functional. In uninfected Arabidopsis plants expressing *LIP5-GFP*, fluorescent signals were observed predominantly in the cytoplasm but also in the nuclei (Figure 10A). To confirm the nuclear localization of LIP5-GFP, we peeled off the leaf epidermal layer of the transgenic plants for DAPI staining and surprisingly found that wounding alters subcellular localization of LIP5 because many fluorescent punctate signals were observed after the leaf epidermis was peeled off (see Figure S12). There was also a 3–5-fold increase in the number of fluorescent punctate signals in the cells after infection with *Pst*DC3000 (Figure 10A). Thus, both wounding and pathogen infection could alter subcellular localization of LIP5.

To examine the effect of the SKD5-LIP5 interaction on the subcellular localization of LIP5, we transiently co-expressed LIP5-GFP with SKD1 in *Nicotiana benthamiana*. Unlike in tobacco leaves expressing only LIP5-GFP, in which the fluorescent signals were largely cytosolic and diffusive as found in Arabidopsis, co-expression of LIP5-GFP with wild-type SKD1 resulted in a large number of punctate fluorescent structures (Figure 10B). No such punctate fluorescent structures were observed when LIP5F395A-GFP was co-expressed with SKD1 in tobacco leaves (Figure 10B). To further analyze this, we co-expressed LIP5-GFP with a mutated version of SKD1, SKD1E232Q, which is unable to hydrolyze ATP and when overexpressed in plant cells induces dominant-negative endosomal sorting defects including enlarged MVBs [12]. Indeed, co-expression of LIP5-GFP with SKD1E232Q led to a substantial number of enlarged, intensely fluorescent punctate structures, concomitant with reduced fluorescent intensity in the cytoplasm in infiltrated tobacco leaf cells (Figure 10B).

**Figure 11:**
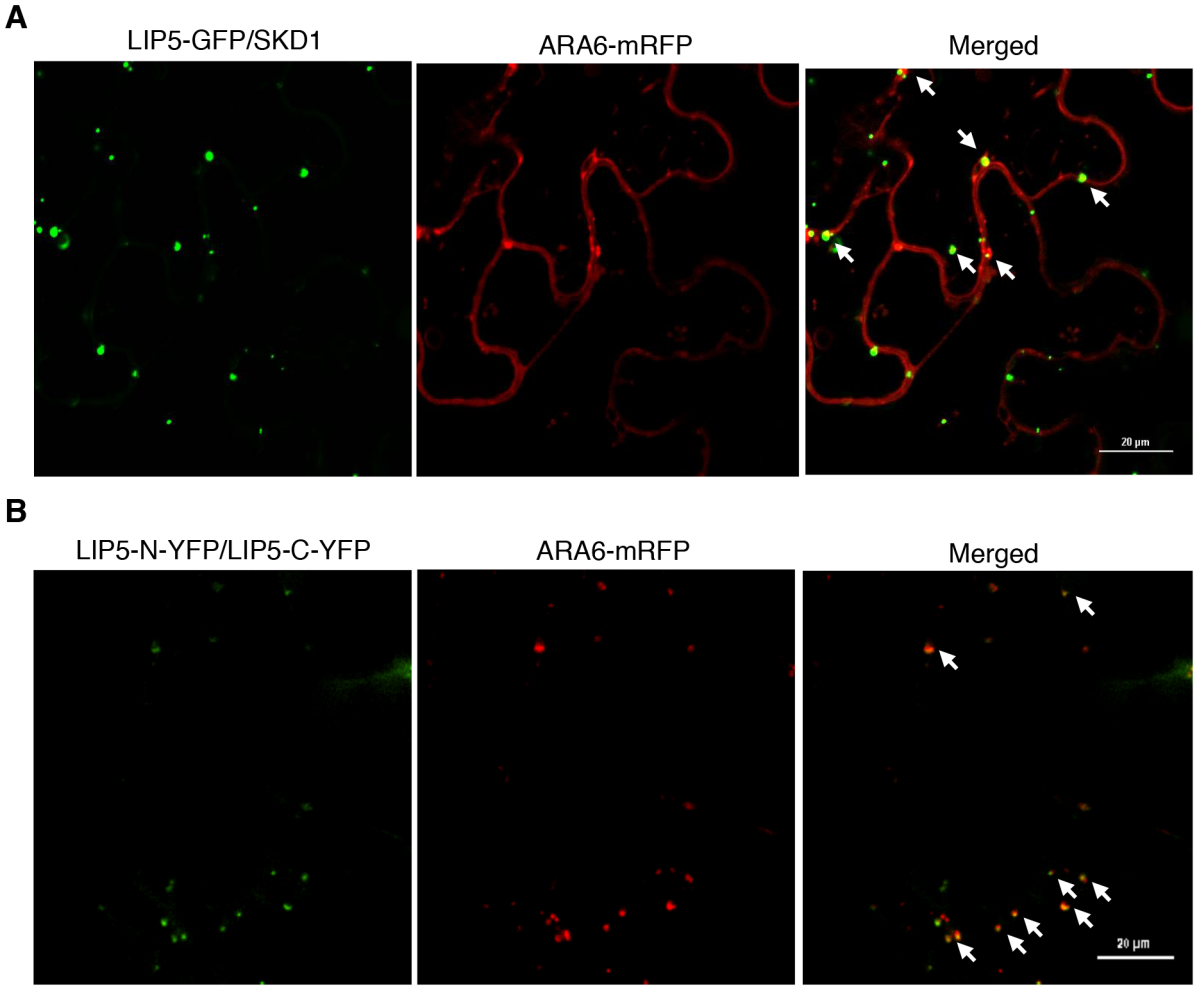
Subcellular colocalization of LIP5 with ARA6-mRFP fusion protein in *N. benthamiana*. (**A**) Partial colocalization of LIP5-GFP with ARA6-mRFP when coexpressed with SKD1 in tobacco epidermal cells. (**B**) Increased colocalization of dimerized LIP5 derived from LIP5-N-YFP/LIP5-C-YFP complementation with coexpressed ARA6-mRFP in tobacco epidermal cells. Arrows indicate punctate areas of colocalization. Bars = 20 µm.

To determine whether the LIP5-GFP punctate signals generated after coexpression with SKD1 are MVBs, we analyzed their colocalization with ARA6 through coexpression of the LIP5-GFP, ARA6-mRFP and SKD1 in *N. benthamiana*. As observed with GFP-SKD1 from a previous study [12], the colocalization analysis between LIP5-GFP and ARA6-mRFP was difficult because of the strong fluorescent signals of LIP5-GFP. Careful examination revealed that about 50% of the LIP5-GFP punctate signals were labeled with ARA6-mRFP (Figure 11A). Interestingly, almost all of the yellow fluorescent punctate signals generated from dimerization of LIP5 through BiFC were labeled with ARA6-mRFP (Figure 11B). This difference in the extent of colocalization of LIP5 and ARA6 could be due to the differential association of the two proteins with MVBs at various stages of biogenesis. It might be possible, for example, that dimerized LIP5 proteins are preferentially associated with ARA6-positive MVBs whereas undimerized LIP5 proteins are associated with ARA6-negative vesicles.

## Discussion

The extensively characterized MPK3/MPK6 signaling cascade plays a crucial role in plant innate immunity [59]. A number of Arabidopsis proteins including WRKY33, ACS2, ACS6 and ERF6 have been identified as substrates of MPK3/6 and functionally analyzed for their roles in plant defense against necrotrophic pathogens [29], [30], [32]. Here, we report that LIP5, a positive regulator of the SKD1 AAA ATPase in MVB pathway, is another substrate of the pathogen-responsive MAPKs and plays an important role in plant basal resistance to the hemibiotrophic bacterial pathogen *P. syringae*. Through comprehensive genetic, molecular and biochemical analysis, we further demonstrated that the critical role of LIP5 in plant basal resistance is dependent on both its interaction with the SKD1 AAA ATPase in MVB biogenesis and its phosphorylation-enhanced stability. Our study provides genetic evidence for the critical role of MVB biogenesis in plant innate immunity and established an important mechanism for the regulation of vesicle trafficking during plant immune responses.

### A Critical Role of LIP5 and LIP5-Regulated MVB Biogenesis in Plant Immunity

It has been reported that the previously isolated *lip5-1* mutant showed no apparent phenotypic defects in normal growth conditions [12]. Although we observed no major phenotypes either with the *lip5-1* mutant, there was a slight but detectable reduction in the size of plants (see Figure S1C). Similar reduction in plant growth was also observed with the *lip5-2* mutant (see Figure S1C). The major phenotype of the *lip5* mutants, however, was increased susceptibility to the bacterial pathogen *P. syringae* (Figure 2 & Figure S3B). Based on both bacterial growth and symptom development, the *lip5* mutants were as susceptible to a virulent strain of the bacterial pathogen as the *sid2* and *npr1* mutants defective in SA biosynthesis and signaling, respectively (Figure 2). These results indicated that while largely dispensable for plant growth and development under normal growth conditions, LIP5 is important in plant immune system. Using a combination of FM1–43 staining, ARA6-GFP imaging and TEM, we further observed that the basal SKD1 activity without stimulating LIP5 is sufficient for the basal endocytic activities in plant cells, consistent with the absence of major growth phenotypes of the *lip5* mutants. However, pathogen-induced endocytic activities were increased in wild-type plants but not in the *lip5-1* mutant plants (Figure 8). This observation suggested that LIP5 is necessary for pathogen-induced vesicle trafficking.

A multitude of plant defense mechanisms including PTI and SA-mediated defense are important in Arabidopsis immune system against *P. syringae* [46], [47]. An increasing number of studies have implicated intracellular protein trafficking in these defense responses. In flg22-triggered immunity, activated pattern-recognition receptor FLS2 undergoes endocytosis upon flg22 binding not only for the attenuation of FLS2 activation but also for signaling required for efficient PTI [15], [16]. In both PTI and SA-mediated defense, Arabidopsis cells rely on various vesicle trafficking pathways for secretion of antimicrobial compounds (e.g. callose) or proteins (e.g. PR proteins) to combat the extracellular bacterial pathogen. A number of plant and pathogen components participate in or affect PTI or SA-mediated defense against *P. syringae* through directing or influencing vesicle trafficking. For example, the AvrPto effector protein from *P. syringae* increases bacterial growth in correlation with suppressed callose deposition and other cell wall-related defense [60]. Genes encoding components in the plant secretory pathway are up-regulated during SA-regulated systemic acquired resistance (SAR) and their mutations compromise SA-induced PR proteins and establishment of SAR [61]. The HopM1 effector protein interact with and cause degradation of the Arabidopsis endosomal protein HopM interactor 7 (AtMIN7), an ADP ribosylation factor quinine nucleotide exchange factor protein that modulates vesicle trafficking and polar deposition of callose in response to bacterial pathogens [62]. AtMIN7 has an important role in both PTI and SA-regulated immunity [63]. Strikingly, despite the high susceptibility to *P. syringae*, the *lip5* mutant plants were responsive to flg22 and SA for induced disease resistance (Figures 6B and 7C). In addition, flg22-induced callose deposition and secretion of SA-induced PR1 were normal in the *lip5* mutants (Figures 6C and 7B). The intriguing phenotypes of the *lip5* mutants suggest that LIP5-regulated MVB pathway plays a distinct role in plant immune responses.

In the post-Golgi endosomal systems of plant cells, there are two types of well-described endosomal compartments, the TGN/early endosome (TGN/EE) and the MVB [2]. Unlike LIP5, which is associated with MVBs, other endosomal proteins including AtMIN7 [62], [63], KEEP ON GOING (KEG) [64], Exo70B2 and Exo70H1 exocyst subunits *(Pecenkova et al., 2011)* and syntaxin SYP132 *(Kalde et al., 2007)* with roles in PTI, ETI and SA-regulated immunity against *P. syringae* localize to the TGN/EE or plasma membrane. TGN/EE is a highly dynamic endosomal compartment that function as a major hub for both secretary and endocytic pathways [2]. In Arabidopsis, pathogen-induced callose is synthesized by PMR4, a plasma membrane integral enzyme that is dependent on the cellular secretory pathway for correct subcellular localization [65], [66]. Likewise, SA-regulated PR proteins enter the secretory pathway for their accumulation in the extracellular space [61], [67]. Mutations of genes encoding TGN/EE- or plasma membrane-localized proteins with critical roles in the secretory pathway would compromise production or secretion of defense-related compounds and proteins for establishment of cell-wall-based defense associated with PTI and SA-regulated SAR. On the other hand, as a regulator of the MVB biogenesis, a late endosomal compartment, LIP5 primarily affects the endocytic pathway. Therefore, critical components of flg22- and SA-induced defense mechanisms such as callose deposition and PR protein secretion may involve primarily secretory pathways that are not subject to regulation by LIP5. Second, LIP5 is a positive regulator, but not an essential component of MVB biogenesis. In the *lip5* mutants, the basal levels of the SKD1 AAA ATP activity is sufficient for normal plant growth and development as indicated from its wild-type growth phenotypes when grown under normal growth conditions [12]. Even if some of the flg22- and SA-mediated defense mechanisms require vesicle trafficking through MVBs, basal SKD1 AAA ATPase activity may be sufficient for the required levels of MVB biogenesis for flg22- and SA-mediated trafficking of defense-related molecules.

Even though the *lip5* mutants were responsive to flg22 and SA, they were hyper-susceptible to *Pst*DC3000 when compared with wild-type plants with or without prior flg22 or SA treatment (Figures 2, [6] and [7]). Thus the role of LIP5-regulated MVB biogenesis in plant immunity is critical and cannot be fully compensated by flg22- or SA-induced defense mechanisms. MVBs contain endocytosed cargoes and deliver them either to vacuolar compartment for degradation or fuse with the plasma membrane to release the internal vesicles (exosomes) into the extracellular space. Indeed, the TEM study revealed that PstDC3000 infection resulted in increased numbers of MVBs and PMBs in wild-type plants and this pathogen-induced MVB/PMB formation was largely LIP5-dependent (Figure 9). A number of molecular processes could take place through activated MVB biogenesis and contribute to plant innate immunity. First, trafficking molecules through late endosomes/MVBs could play a direct role in plant immune systems by executing a timely, focal defense response to invading pathogens. In cereal plants attacked by powdery mildew fungal pathogens, penetration resistance is conferred by local cell wall appositions (papillae) deposited by the epidermal cells between the cell wall and plasma membrane [21]. Papillae contain callose and extracellular membrane materials and it has been suggested that these defense-related materials are loaded via MVBs into barely papillae based primarily on the accumulation of MVBs around papillae and presence of extracellular exosome-like vesicles beneath sites of fungal attack [19],[20],[22]. In spite of extensive microscopic results, there has been no direct genetic evidence for a critical role of MVBs in plant immune responses as mutations of genes essential for MVB biogenesis are often lethal [12], [25]. The demonstrated role of LIP5, an established regulator of MVB biogenesis, in plant basal resistance to *P. syringae* (Figure 2) and in pathogen-induced MVB/PMB formation (Figure 9) strongly suggests that reorganization of defense-related materials via MVBs may play a critical role in focally directing extracellular immune responses to invading pathogens including bacterial pathogens. Second, internalization of ligand-activated plasma membrane receptors by endocytosis and their localization to MVBs en route to vacuoles can lead to attenuation, stabilization or even stimulation of signaling [68]. Arabidopsis contain a large number of membrane receptors including more than 600 transmembrane receptor-like proteins (RLKs) with versatile N-terminal extracellular domains and C-terminal intracellular kinases [69]. These RLKs participate in regulatory signaling of plant growth, development and stress responses and may influence, directly or indirectly, plant immune responses in broad and diverse manners. Pathogen-induced endocytosis of membrane receptors may, therefore, constitute part of the global signaling reprogramming required for effective defense responses. Third, the composition of integral membrane proteins including nutrient transporters, ion channels and signaling receptors is critical for the growth, differentiation and survival of eukaryotic cells. As a result, the complex process of remodeling cell surface protein composition is necessary, particularly under stress conditions [70]. Removal of nutrient transporters by pathogen-induced endocytosis and MVB biogenesis, for example, may result in reduced export of necessary nutrients for the growth of extracellular pathogens. The structures and functions of integral membrane proteins can also be altered or damaged in pathogen-infected plants. Pathogen-induced endocytosis and MVB biogenesis can act as an important quality control mechanism that removes damaged and potentially toxic integral membrane proteins to promote health of plant host cells. The isolated *lip5* mutants will be valuable for identifying and dissecting the mechanisms by which defective in pathogen-induced endocytosis and MVB biogenesis contribute to plant immune responses.

### Regulation of LIP5 Stability and MVB Biogenesis by Pathogen-Responsive MAPKs

LIP5 interacted with MPK6 (Figure 1) and probably with MPK3 as well [37]. Furthermore, phosphorylation of LIP5 by both MPK3 and MPK6 was demonstrated based on three different types of assays. Recombinant LIP5^WT^ but not LIP5^6A^ could be phosphorylated *in vitro* by activated recombinant MPK6 and MPK3 (Figure 3A). Recombinant LIP5 could also be phosphorylated *in vitro* by native MPK3 and MPK6 from flg22-treated plants (Figure 3B). In addition, myc-tagged LIP5^WT^ but not LIP5^6A^ expressed in transgenic plants underwent increased phosphorylation *in vivo* upon pathogen infection or upon induced expression of gain-of-function NtMEK2^DD^ (Figure 4), which phosphorylates and activates Arabidopsis MPK3 and MPK6 [71]. In these transgenic plants, importantly, LIP5^WT^ accumulated to higher levels than LIP5^6A^ even in uninfected plants and the difference became more pronounced following activation of the MPKs through induced expression of gain-of-function NtMEK2^DD^ or pathogen infection (Figure 4). The differential accumulation of myc-LIP5^WT^ and myc-LIP5^6A^ was correlated with the differential phosphorylation of the expressed LIP5 proteins (Figure 4). These results indicated that phosphorylation of LIP5 by the pathogen-responsive MPKs increased its stability. Similarly, phosphorylation of Arabidopsis ACS2, ACS6 and ERF6 by MPK3/MPK6 increase the stability of the substrates (Li et al., 2012; Meng et al., 2013). Therefore, phosphorylation-dependent regulation of protein substrate stability appears to be a common mechanism through which MPK3/MPK6 regulates plant stress and defense responses.

LIP5^6A^ was as active as LIP5^WT^ in interaction with both SKD1 and MPK6 (see Figure S6). Furthermore, the phosphorylation mutant LIP5^6A^ was fully capable of complementing the *lip5-1* mutant for disease resistance when it was expressed at high levels in the transgenic plants (Figure 5). By contrast, mutant LIP5F395A incapable of interacting with SKD1 failed to complement the *lip5-1* mutant plant even when it was expressed at high levels (Figure 2C & 2D). These results strongly suggested that phosphorylation of LIP5 by the pathogen-responsive MPKs affects primarily the stability but not the other critical properties of LIP5. When expressed at medium or low levels, LIP5^WT^ but not LIP5^6A^ was fully able to complement the *lip5-1* mutant for resistance to *Pst*DC3000, in correlation with the differential accumulation of the LIP5^WT^ and LIP5^6A^ proteins in the transgenic plants (Figure 5). Thus, the protein level of LIP5^6A^ in the transgenic *lip5* mutant plants is the primary determinant for its ability to complement the *lip5* mutant phenotypes.

Expression of *LIP5* appeared to be relatively low based on RNA blot analysis of its transcripts in both healthy and pathogen-infected plants (see Figure S10). The expression of *LIP5* also appeared to be under control of not only its promoter but also its other noncoding sequences because we failed to detect accumulation of either the *LIP5* transgene transcripts or the gene product when a myc-tagged *LIP5* transgene under control of its native promoter was transformed into the *lip5* mutant plants. As a result, we used the constitutive CaMV *35S* promoter in the *LIP5* expression constructs and relied on the variation in transgene expression levels for testing the effects of protein phosphorylation on LIP5 protein accumulation and its ability to complement the *lip5* mutant phenotypes (Figure 5A). To ensure that the results with the CaMV *35S* promoter are physiologically relevant, we compared the levels of the *LIP5^WT^* and *LIP5^6A^* transgene transcripts in the *lip5* mutant background with those the native *LIP5* gene in pathogen-infected wild-type plants. These experiments showed that expression level of the native *LIP5* was even lower than those of the *LIP5^WT^* and *LIP5^6A^* transgenes in the *lip5-1/35S::LIP5^WT^-L* and *lip5-1/35S::LIP5^6A^-L* lines (see Figure S8). Accordingly, we expect that the stability and, consequently, the protein level of the native LIP5 will be even more strongly affected by protein phosphorylation than the myc-tagged LIP5 proteins in the *lip5-1/35S::LIP5^WT^-L* and *lip5-1/35S::LIP5^6A^-L* lines. Therefore, the levels of LIP5 appear to be under stringent control at multiple levels. In healthy plants, the levels of LIP5 are maintained at low levels through low expression. Upon pathogen infection, the LIP5 levels are elevated by significantly elevated expression and, more importantly, increased stability through protein phosphorylation. Interestingly, constitutive expression of high levels of LIP5 or induced expression of the gain-of-function *NtMEK2^DD^* without pathogen infection did not lead to constitutive induction of MVB formation (data not shown), indicating that phosphorylation-dependent LIP5 stability is necessary but not sufficient for increased MVB formation.

In summary, we have demonstrated that Arabidopsis LIP5, a positive regulator of MVB biogenesis, is a critical target of pathogen-responsive MPK3/6 cascade in plant basal defense. LIP5 is expressed at very low levels in healthy plants but its protein levels can be substantially elevated through phosphorylation by pathogen-responsive MPKs to promote pathogen-regulated vesicle trafficking. Disruption of the *LIP5* gene compromised pathogen-induced MVB and PMB formation and rendered plants highly susceptible to *P. syringae*. The critical role of LIP5 in plant basal resistance is dependent on both its ability to interact with SKD1 and its increased stability through protein phosphorylation. Despite their high susceptibility to *P. syringae*, the *lip5* mutants were responsive to flg22 and SA and were normal in flg22- and SA-induced disease resistance, indicating that LIP5-regulated MVB pathway plays a critical and unique role in plant immune system.

## Methods

### Arabidopsis Genotypes and Plant Growth Conditions

The Arabidopsis mutants and wild-type plants used in the study are all in the Col-0 background. The *lip5-1* and *npr1-3* mutants have been previously described [12], [38]. Homozygous *lip5-2* (GABI_315F05), *sid2-3* (Salk_133146), *fls2-2* (Salk_062054), *mpk3* (Salk_151594) and *mpk6* (Salk_062471) mutants were identified by PCR using primers flanking the T-DNA insertions listed in Table S1. Arabidopsis and *N. benthemiana* plants were grown in growth chambers at 22°C, 120 µE m^−2^ light on a photoperiod of 12-hour light and 12 h dark.

### Yeast Two-Hybrid Screen and Assays

To identify MPK-Interacting proteins, we used the Gal4 based yeast-two-hybrid system as described by the manufacturer (Stratagene). Full-length *MPK6* sequence was PCR-amplified using gene-specific primers (5′-atcgtcgacatggacggtggttcaggt-3′ and 5′-atcgtcgacctattgctgatattctggattgaaa-3′) and cloned into pBD-GAL4 vector to generate the bait vector. The *Arabidopsis* HybridZAP-2.1 two-hybrid cDNA library was prepared from *Arabidopsis* plants as previously described [72]. The bait plasmid and the cDNA library were used to transform yeast strain YRG-2. Yeast transformants were plated onto selection medium lacking Trp, Leu and His and confirmed by β-galactosidase activity assays using ONPG (o-nitrophenyl-β-D-galactopyranose) as substrate.

For assaying SKD1-LIP5 or LIP5-LIP5 interactions in yeast cells, full-length *SKD1* and *LIP5* coding sequences were PCR-amplified using gene-specific primers (*SKD1*: 5′-agcgaattcatgtacagcaatttcaaggaac-3′ and 5′-agcctcgagtcaaccttcttctccaaactcc-3′; *LIP5*: 5′-atgcgaattcatgtcgaacccaaacgaac-3′ and 5′-agctgtcgactcagtgaccggcaccggccga-3′) and cloned into pBD-GAL4 or pAD-GAL4 vectors as appropriate. Various combinations of bait and prey constructs were cotransformed into yeast cells and interactions were analyzed by assaying *LacZ* β-galactosidase activity.

### BiFC Assay

The BiFC vectors pFGC-N-YFP and pFGC-C-YFP have been previously described [73]. The full-length *LIP5*, *MPK3* and *MPK6* sequences were PCR-amplified using gene-specific primers (*LIP5*: 5′-agctctagatctatgtcgaacccaaacga-3′ and 5′-atcggatccgtgaccggcaccggccga-3′; *MPK3*: 5′-atcggatccatgaacaccggcggtgg-3′ and 5′-atctctagaaccgtatgttggattgagtgct-3′; *MPK6*: 5′-atcggatccatggacggtggttcaggt-3′ and 5′-atctctagattgctgatattctggattgaaagca-3′) and cloned into pFGC-C-YFP or pFGC-N-YFP, as appropriate. The plasmids were introduced into *Agrobacterium tumefaciens* (strain GV3101) and transformed into Arabidopsis plants. Positive T1 transformants were identified by RNA blotting and crossed to generate transgenic plants containing a pair of BiFC constructs. Fluorescence and DAPI were visualized under a Zeiss LSM710 confocal microscope and images were superimposed with Zeiss LSM710 software.

### Quantitative RT-PCR

Total RNA isolation and quantitative real-time PCR analysis of *LIP5* transcripts in the *lip5* mutants was performed using *LIP5*-specific primers (5′-aggctgctagattcgctgtg-3′ and 5′-ggccgatggatttgttagc-3′) and Arabidopsis *ACTIN2* gene was used as internal control as previously described [74].

### Pathogen Inoculation and flg22 Treatment

*Pst*DC3000 inoculation was performed as previously described [40]. For tittering bacteria, inoculated leaves were homogenized in 1 ml 10 mM MgCl_2_ and diluted before plating on King's B Agar with appropriate antibiotics. Colony forming units were counted 2–3 days after bacteria growth at room temperature. Chlorophyll contents of inoculated leaves were determined as previously described [75]. Analysis of hypersensitive cell death after infection of avirulent strains of *Pst*DC3000 including trypan blue staining was performed as previously described [40], [76].

The flg22 peptide was first dissolved in water to make a 10 mM stock solution. For testing flg22-induced growth inhibition, seeds of different genotypes were germinated and grown on ½ MS agar for 5 days before moving to ½ MS liquid medium containing flg22 at various concentrations in 12-well plates. Seedlings were further grown for 7 days before measuring seedling fresh weight. For determining flg22-induced disease resistance, 1 µM flg22 was pre-infiltrated into 3 leaves per plant one day before pathogen infection. For examining flg22-induced callose deposition, 10-days old Arabidopsis seedlings were transferred to ½ MS liquid medium containing 0, 0.2 or 1 µM flg22. Seedlings were stained by aniline blue 24 hours later as previously described [77]. Briefly, leaves were cleared with alcoholic lacto-phenol to remove chlorophyll, and washed first in 50% ethanol and then in water. Leaves were stained in a solution containing 0.01% aniline blue (Sigma-Aldrich) in 150 mM K_2_HPO_4_, pH 9.5 in dark for 30 min before being mounted in 50% glycerol. Pictures were taken with Nikon eclipse E800 epifluorescence microscopy and callose deposition was quantified from more than 10 125×100 µm microscopic fields per treatment per genotype. For analyzing flg22-induced defense genes, plants were treated with H_2_O_2_ or 100 nM flg22 and the samples collected after 30-minute treatment were used for analysis of *WRKY* gene expression and samples collected at 24-hour treatment were used for analysis of *PR* gene expression.

### Generation of Mutant *LIP5* and *SKD1* Genes

Mutant genes for SKD1E232Q, LIP5F388A, LIP5F395A, LIP5F388A/F395A and LIP5^6A^ were generated by QuickChange site-directed mutagenesis (Stratagene) or overlapping PCR using the primers listed in Table S2. The mutations were confirmed by DNA sequencing.

### Generation of Transgenic *myc-LIP5* Lines

To generate myc-tagged wild-type and mutant *LIP5* genes for overexpression in Arabidopsis, we first amplified these *LIP5* coding sequences using a pair of *LIP5*-specific primers (5′-gcacatatgtcgaacccaaacgaacca-3′ and 5′-atcggatcctcagtgaccggcaccggccga-3′). The amplified *LIP5* fragments were fused with a 4xmyc tag sequence in a modified pBlueScript vector [30]. The myc-tagged wild-type and mutant *LIP5* genes were then subcloned into a modified pBI121 binary vector [30]. The resulting constructs were introduced into the *Agrobacterium tumefaciens* (strain GV3101) and transformed into Arabidopsis by floral dip method [78]. Transformants were identified by kanamycin resistance and expression of the transgenes was analyzed by RNA blotting or immunoblotting using an anti-myc monoclonal antibody (Sigma-Aldrich).

### RNA Gel Blot Analysis

Total RNA isolation, separation and blot analysis using ^32^P-labeled DNA probes were performed as previously described [79].

### Immunoblotting

Protein extraction, separation and blot analysis were performed as previously described [80]. The protein concentration was determined using the Bio-Rad protein assays kit using BSA as standard. Detection of myc-tagged proteins was performed using an anti-myc monoclonal antibody (Sigma-Aldrich).

### Analysis of PR1 in Intercellular Wash Fluid (IWF)

A 1.5 kb promoter sequence of Arabidopsis *PR1* gene was PCR-amplified using the following primers (5′-gttagcacaagcttgttttaacttataaaa-3′ and 5′-atcggatccttttctaagttgataatggttattgttgtg-3′) and cloned into a modified pCAMBIA1300p plant transformation vector. Tobacco acidic *PR1a* coding sequence was PCR amplified using gene-specific primers (5′-agcccatgggatttgttctcttttcaca-3′ and 5′-agctctagattagtatggactttcgcctct-3′) and placed behind the Arabidopsis *PR1* promoter. The resulting construct, designated as *P_AtPR1_::NtPR1* was transformed into Arabidopsis plants. Preparation of total proteins and IWF were performed as previously described (Wang et al., 2005). Immunoblot detection of NtPR1 was performed using the 33G1 monoclonal antibody against tobacco PR1 (Chen et al., 1993). Possible contamination of intracellular proteins in IWF was examined using an anti-catalase monoclonal antibody [81].

### Confocal Imaging of FM1–43 Internalization and Staining

FM1–43 (Sigma-Aldrich) was dissolved in H_2_O as a 4 mM stock solution. Arabidopsis leaves were cut into smaller pieces and stained in 20 µM FM1–43 for 1 hour at room temperature, washed and examined under a Zeiss LSM710 confocal microscope. Internalized fluorescent puncta were identified as endocytic vesicles and counted from more than 20 60×60 µm microscopic fields per treatment per genotype. Full-length *ARA6/RabF1* gene was PCR-amplified using the ARA6-specific primers (5′-agcgaattcatgggatgtgcttcttctct-3′; 5′-agcggatcctgtgacgaaggagcaggacgag-3;) and fused to the GFP gene in a binary plant transformation vector and transformed into Arabidopsis plants. The transgenic Arabidopsis plants were used for imaging under a confocal microscope following *Pst*DC3000 infection and stained with FM1–43 for analysis of colocalization of FM1–43 and ARA6-GFP. Because of significant overlap between the FM1–43 and GFP fluorophores, colocalization of ARA6-GFP and FM1–43 staining was done by fluorescence unmixing. Briefly, FM1–43 stained ARA6-GFP transgenic plant leaves were excited at 488 nm. Emission was collected by the use of 15-channel fluorescence imaging, each channel encompassing 10 nm wide from 500 nm to 650 nm. Fluorescence unmixing of the image data was performed using Spectral Unmixing Tools with GFP-only spectrum and FM1–43-only spectrum (lipid) as references for the reliable separation of overlapping fluorescence signals and colocalization analysis.

### Transmission Electron Microscopy (TEM)

The electron microscope was done at the Purdue Bindley Bioscience Center - Imaging Facility. Samples were fixed by the microwave under low vacuum. Briefly, leaf samples were cut into pieces 1–2 mm long and fixed with 2% paraformaldehyde (v/v) and 2.5% glutaraldehyde (v/v) in 0.1 M cacodylate buffer, pH 6.8, and rinsed with 0.1 M cacodylate buffer, pH 6.8. Tissues were further treated with 1% OsO_4_ (v/v) and 1.5% K_3_Fe(CN)_6_ (v/v) and washed. The samples were then dehydrated in a grade ethanol series and 100% propylene oxide at final change. Infiltration was done on the bench by 25% Spurr (overnight) and 50% (without accelerator), 75% Spurr with Accelerator (overnight) and 100% Spurr for 6 hours. Tissues were embedded in a flat mold with resin and polymerized at 60°C for 48 hours.

Samples were cut with a diamond knife using an ultramicrotome (LEICA EM UC6), sections were collected on copper grids coated with formvar and carbon, then poststained 5 min with 2% uranyl acetate in 70% methanol and Reynold's lead citrate for 3 min. Samples were imaged using a Philips CM-100 TEM (FEI Company, Hillsboro, OR) operated at 100 kv, spot 1, 200 µm condenser aperture and 70 µm objective aperture. Images were captured on an SIA L3-C digital camera. MVBs and PMBs were manually quantified from randomly selected cells in each genotype/treatment.

### Subcellular Localization

Full-length *LIP5* gene was fused to the *GFP* gene behind the CaMV *35S* promoter in a modified pCAMBIA1300 plant transformation vector and transformed into Arabidopsis plants. Standard confocal laser microscopy of stably transformed Arabidopsis leaves was performed for imaging of GFP.

For transient co-expression of *LIP5-GFP* with *SKD1* and *SKD1E232Q* in *N. benthamiana*, the wild-type and mutant *SKD1* genes was PCR-amplified using *SKD1*-specific primers (5′-agcctcgagatgtacagcaatttcaaggaac-3′ and 5′-agctctagatcaaccttcttctccaaactcc-3′) and cloned into pTA7002 under control of a dexamethasone (DEX)-inducible promoter. *Agrobacterium* cells containing *LIP5-GFP* and *SKD1* constructs to be co-expressed were co-infiltrated into *N. benthamiana* leaves. Two days after infiltration, the leaves were infiltrated with 30 µM DEX to induce *SKD1* expression and imaging of GFP was examined under a Nikon eclipse E800 epifluorescense microscope one day after DEX treatment.

For colocalization analysis of LIP5 with MVB marker ARA6, *LIP5-GFP* and *SKD1* transgenes were coexpressed with the *ARA6-mRFP* marker gene [12] in *N. benthamiana*. Dimerized LIP5 fluorescent signals were generated from complementation of coexpressed LIP5-N-YFP and LIP5-C-YFP constructs in *N. benthamiana*. Imaging of coexpressed GFP, YFP and mRFP signals was performed with standard confocal laser microscopy.

### Preparation of Recombinant Proteins, *In Vitro* Phosphorylation and *In-Gel* Kinase Assays

Full-length *LIP5^WT^* and *LIP5^6A^* coding sequences were PCR amplified and cloned into pET32a vector. Preparation of recombinant LIP5 and the in vitro phosphorylation assay were performed as previously described [30].

For preparation of native MPK3 and MPK6, total protein extracts were isolated from flg22-treated seedlings. In gel kinase assay was performed as previously described [30], using recombinant LIP5 proteins as substrates.

### *In Vivo* Phosphorylation of LIP5

For examining phosphorylation of LIP5 by pathogen-responsive MPK3/6, which are activated by the gain-of-function NtMEK2^DD^, transgenic Arabidopsis harboring a *35S::myc-LIP5* construct was crossed with transgenic lines containing *NtMEK2^DD^* driven by a DEX-inducible promoter in pTA7200 [42], [82]. Progeny plants containing both constructs were examined for *myc-LIP5* gene expression and assays of *in vivo* phosphorylation as previously described. Phos-tag Acrylamide (NARD Institute) was used for phospho-protein mobile shift assay to detect *in vivo* phosphorylation of LIP5 protein. Briefly, total proteins were separated in a 10% SDS-PAGE gel containing 100 µM Phos-tag and 200 µM MnCl_2_. myc-LIP5 protein shifts were detected by western blotting with anti-myc antibody.

For dephosphorylation assays, protein extracts were isolated from DEX-treated transgenic *NtMEK2^DD^/myc-LIP5^WT^* or pathogen-infected *lip5-1*/*myc-LIP5^WT^* plants and treated at 37°C for 45 minutes with CIP (0.4 U/µl) in the absence or presence of phosphatase inhibitors (10 mM NaF, 7 mM β-glycerophosphate and 5 mM Na-pyrophoshate). The protein extracts were subsequently separated on the regular SDS-PAGE and Phos-tag gels for immunoblot analysis using an anti-myc monoclonal antibody.

### Accession Numbers

Arabidopsis Genome Initiative numbers for the genes discussed in this article are as follows: MPK3 (At3g45640), MPK6 (At2g43790), LIP5 (At4g26750), NPR1 (At1g64280), SID2 (At1g74710), FLS2 (At5g46330), PR1 (At2g14610) and SKD1 (At2g27600).

## Supporting Information

Figure S1 Identification of *lip5* mutants. (**A**) Diagram of the *LIP5* gene and the insertion sites of the *lip5-1* and *lip5-2* mutants. (**B**) Transcript levels of *LIP5* in Col-0 wild type (WT) and *lip5* mutants as determined using real-time qRT-PCR. Error bars indicate SE (n = 3). (**C**) Mature Plants of Wild Type (WT), *lip5* Mutants and *lip5-1* Complemented with the *myc-LIP5* Transgene. The picture was taken about six weeks after germination. The *lip5* mutant plants are slightly but significantly smaller than WT and complemented *lip5* line.
(PDF)

Figure S2 Effects of *Pst*DC3000 infection on chlorophyll contents of infected leaves. (**A**) Chlorophyl contents of infected leaves of Wild-type (WT), *lip5*, *npr1* and *sid2* mutant plants. Plants were infiltrated with a suspension of the virulent *Pst*DC3000 strain (OD_600_ = 0.0002 in 10 mM MgCl_2_). Samples were taken at 0 and 4 dpi for determination of chlorophyll content. The means and standard errors were calculated from 10 plants for each mutant. According to Duncan's multiple range test (P = 0.05), chlorophyll contents do not differ if they are indicated with the same letter. (**B**) Chlorophyll contents of infected leaves of WT, *lip5-1* and *lip5-2* mutant plants complemented with wild-type or mutant *LIP5* genes. Pathogen inoculation and chlorophyll content determination were performed as in **A**.
(PDF)

Figure S3 Responses to avirulent *Pst*DC3000 strains. (**A**) Pathogen-induced hypersensitive cell death. One half of the wild-type (WT), *lip5* and *npr1* mutant leaves was infiltrated with a suspension of an avirulent *Pst*DC3000 strain (OD_600_ = 0.1 in 10 mM MgCl_2_). Representative inoculated leaves were photographed (upper panel) or subjected to trypan blue staining (lower panel) at the indicated hours post inoculation (hpi). (**B**) Enhanced susceptibility of the *lip5-1* Mutant to avirulent *Pst*DC3000 strains. WT and *lip5-1* mutant plants were infiltrated with a suspension of an avirulent *Pst*DC3000 strain (OD_600_ = 0.0002 in 10 mM MgCl_2_). Samples were taken at 0 and 5 dpi to determine the growth of the bacterial pathogen. The means and standard errors were calculated from 10 plants for each mutant. According to Duncan's multiple range test (P = 0.05), means of colony-forming units (cfu) do not differ if they are indicated with the same letter.
(PDF)

Figure S4 LIP5 protein sequences. (**A**) Alignment of C-terminal domains of LIP5 proteins from yeast, human and Arabidopsis. The conserved tyrosine (Y) and phenylalanine (F) residues critical for interaction with SKD1 are indicated in red. (**B**) Arabidopsis LIP5 protein sequence. The six putative MPK phosphorylation sites are indicated in red.
(PDF)

Figure S5 Yeast two-hybrid assays of mutant LIP5 protein interactions. (**A**) Yeast two-hybrid assays of LIP5-SKD1 interaction. Full-length *LIP5*, *LIP5F388A*, *LIP5F395A* and *LIP5F388A/F395A* coding sequences were introduced into the pAD-Gal4 prey vector and cotransformed with the pBD-SKD1 fusion bait vector into yeast cells. Empty pAD-Gal4 vector was used as negative prey control (−). Yeast transformants were analyzed for *LacZ* reporter gene expression through assays of β-galactosidase activity using ONPG as a substrate. Five separate colonies per construct were used for assays of LacZ β-galactosidase activity. (**B**) Yeast two-hybrid assays of LIP5 dimerization. Assays with indicated prey and bait vectors were performed as in **A**.
(PDF)

Figure S6 Normal interaction of LIP5^6A^ with MPK6 and SKD1 in yeast cells. Full-length *LIP5^6A^* coding sequence was introduced into the pAD-Gal4 prey vector and was cotransformed with an empty bait vector (−) or with the pBD-MPK6 and pBD-SKD1 fusion bait vectors into yeast cells. Yeast transformants were analyzed for *LacZ* reporter gene expression through assays of β-galactosidase activity using ONPG as a substrate. Five separate colonies per construct were used for assays of LacZ β-galactosidase activity.
(PDF)

Figure S7 Dephosphorylation of *in vivo* phosphorylated LIP5 proteins. Protein extracts were isolated from transgenic *NtMEK2^DD^/myc-LIP5^WT^* at 24 hours after DEX treatment (**A**) or *lip5-1*/*myc-LIP5^WT^* (**B**) plants at 24 hpi of *Pst*DC3000. The protein extracts was treated at 37°C for 45 minutes with calf intestinal alkaline phosphatase (CIP) in the absence or presence of a phosphatase inhibitor cocktail (10 mM NaF, 7 mM β-glycerophosphate and 5 mM Na-pyrophoshate). Reactions without CIP and phosphatase inhibitors (−) were used as control. The protein extracts were subsequently separated on the regular SDS-PAGE and Phos-tag gels for immunoblot analysis using an anti-myc monoclonal antibody. Rubisco staining of the regular SDS-PAGE gel was used for assessing equal protein loading.
(PDF)

Figure S8 Comparison of the transcript levels of the native *LIP5* gene and the *myc-LIP5* transgenes. Total RNA was isolated from Col-0 wild-type (WT) plants and *lip5-1/myc-LIP5^WT^* and *lip5-1/myc-LIP5^6A^* lines each with similarly high (H), medium (M) and low (L) levels of *myc-LIP5* transcripts and probed with a ^32^P-labeled *LIP5* DNA fragment. Ethidium bromide staining of rRNA was shown for the assessment of equal loading.
(PDF)

Figure S9 Flg22-induced expression of *WRKY* and *PR* genes. Col-0 wild-type (WT) and *lip5* mutant plants were treated with H_2_O_2_ (−) or 100 nM flg22. Total RNA was isolated from samples collected after 30-minute treatment for analysis of *WRKY* gene expression or 24-hour treatment for analysis of *PR* gene expression. RNA blotting analysis of *WRKY* and *PR* gene expression was performed using ^32^P-labeled gene probes. Ethidium bromide staining of rRNA is shown for the assessment of equal loading.
(TIF)

Figure S10 Expression of *LIP5* and *SKD1* in response to pathogen infection. Col-0 wild-type plants were infiltrated with 10 mM MgCl_2_ (mock) or *Pst*DC3000 (OD_600_ = 0.0002 in 10 mM MgCl_2_). Samples were collected at indicated days post-inoculation (dpi) for total RNA isolation and RNA blotting analysis of *LIP5* and *SKD1* gene expression using ^32^P-labeled gene probes. Ethidium bromide staining of rRNA is shown for the assessment of equal loading.
(PDF)

Figure S11 Activity of LIP5-GFP in plant disease resistance. (**A**) Western blotting analysis of LIP5-GFP fusion protein. Total proteins were isolated from untransformed Arabidopsis (−) or transgenic LIP5-GFP plants and subjected to western blot analysis using an anti-GFP monoclonal antibody. The antibody detected a protein band with molecular mass expected to be that of LIP5-GFP from transgenic LIP5-GFP plants. Other proteins detected by the antibody due to nonspecific binding are present in both untransformed Arabidopsis and transgenic *LIP5-GFP* plants. (**B**) Disease symptom development. Wild type (WT), *lip5-1* and *lip5-1/LIP5-GFP* plants were infiltrated with a suspension of *Pst*DC3000 (OD_600_ = 0.0002 in 10 mM MgCl_2_). Pictures of representative inoculated leaves taken at 4 dpi. (**C**) Bacterial growth. Pathogen inoculation of wild-type and mutant plants was performed as in **A**. Samples were taken at 0 or 4 dpi to determine the bacterial growth. The means and standard errors were calculated from 10 plants for each mutant. According to Duncan's multiple range test (P = 0.05), means of colony-forming units (cfu) do not differ if they are indicated with the same letter.
(PDF)

Figure S12 Wound-induced alteration in subcellular localization of LIP5-GFP in leaf epidermal cells. Leaf epidermal layer of transgenic Arabidopsis plants expressing *LIP5-GFP* was peeled off, stained in a DAPI solution and observed under a confocal microscope. Bars = 10 µm.
(PDF)

Table S1 Primers for screening of T-DNA insertion mutants.
(PDF)

Table S2 Primers for construct mutant *LIP5* and *SKD1* genes.
(PDF)
