# Supplementary material for: Arabidopsis LIP5, a Positive Regulator of Multivesicular Body Biogenesis, Is a Critical Target of Pathogen-Responsive MAPK Cascade in Plant Basal Defense
Source: PLoS Pathog. 2014 Jul 10;10(7):e1004243. doi: 10.1371/journal.ppat.1004243 (PMC4092137; doi:10.1371/journal.ppat.1004243)
Supplement: Figure S1 — Identification of lip5 mutants. (A) Diagram of the LIP5 gene and the insertion sites of the lip5-1 and lip5-2 mutants. (B) Transcript levels of LIP5 in Col-0 wild type (WT) and lip5 mutants as determined using real-time qRT-PCR. Error bars indicate SE (n = 3). (C) Mature Plants of Wild Type (WT), lip5 Mutants and lip5-1 Complemented with the myc-LIP5 Transgene. The picture was taken about six weeks after germination. The lip5 mutant plants are slightly but significantly smaller than WT and complemented lip5 line. (PDF) [file ppat.1004243.s001.pdf]

Figure S1

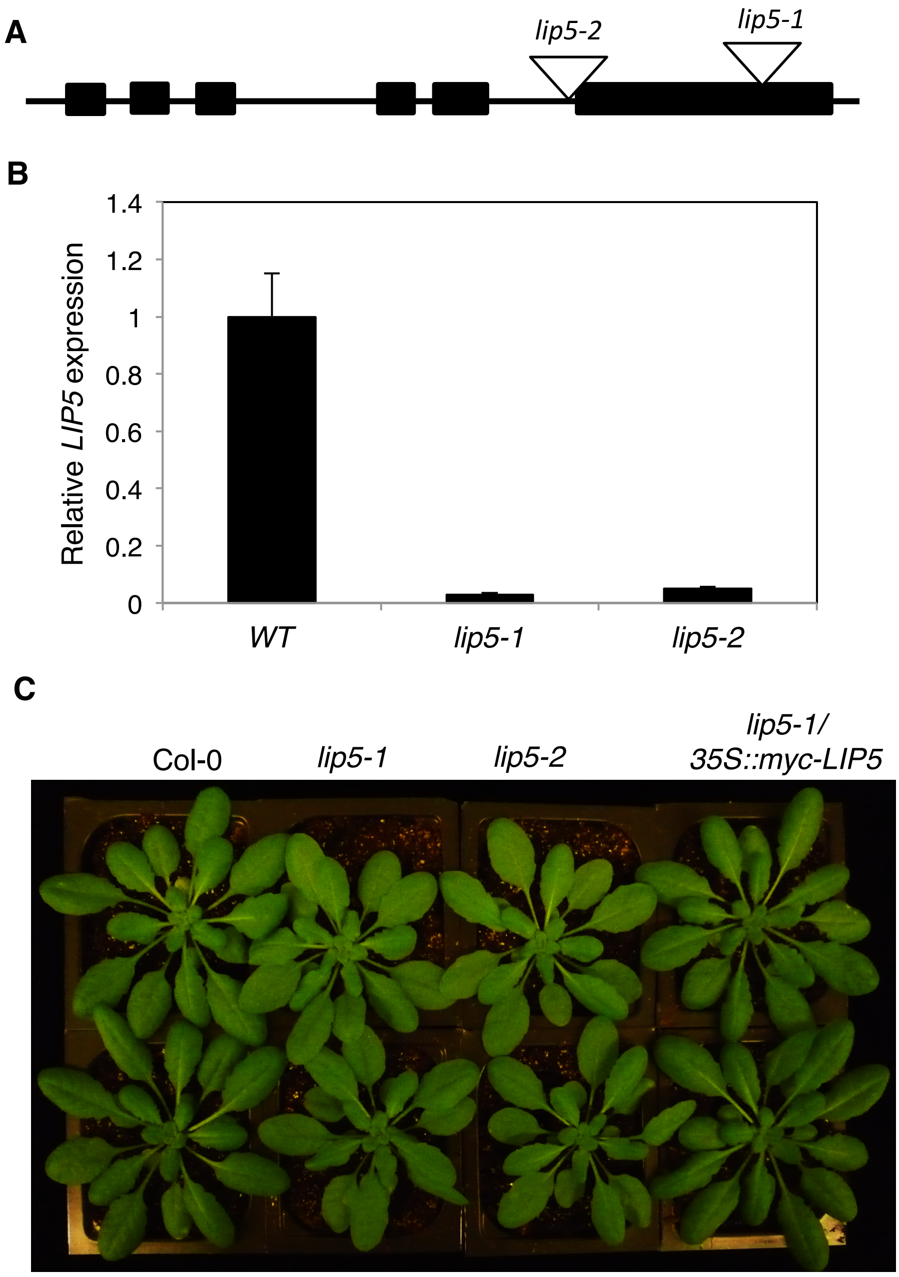
A
lip5-2
lip5-1
B
Relative LIP5 expression
1.4
1.2
1
0.8
0.6
0.4
0.2
0
WT
lip5-1
lip5-2
C
Col-0
lip5-1
lip5-2
lip5-1/
35S::myc-LIP5

Figure S1. Identification of *lip5* mutants

(A) Diagram of the *LIP5* gene and the insertion sites of the *lip5-1* and *lip5-2* mutants.

(B) Transcript levels of *LIP5* in Col-0 wild type (WT) and *lip5* mutants as determined using real-time qRT-PCR. Error bars indicate SE (n=3).

(C) Mature Plants of Wild Type (WT), *lip5* Mutants and *lip5-1* Complemented with the *myc-LIP5* Transgene. The picture was taken about six weeks after germination. The *lip5* mutant plants are slightly but significantly smaller than WT and complemented *lip5* line.
