# Supplementary material for: Arabidopsis LIP5, a Positive Regulator of Multivesicular Body Biogenesis, Is a Critical Target of Pathogen-Responsive MAPK Cascade in Plant Basal Defense
Source: PLoS Pathog. 2014 Jul 10;10(7):e1004243. doi: 10.1371/journal.ppat.1004243 (PMC4092137; doi:10.1371/journal.ppat.1004243)
Supplement: Figure S2 — Effects of PstDC3000 infection on chlorophyll contents of infected leaves. (A) Chlorophyl contents of infected leaves of Wild-type (WT), lip5, npr1 and sid2 mutant plants. Plants were infiltrated with a suspension of the virulent PstDC3000 strain (OD600 = 0.0002 in 10 mM MgCl2). Samples were taken at 0 and 4 dpi for determination of chlorophyll content. The means and standard errors were calculated from 10 plants for each mutant. According to Duncan's multiple range test (P = 0.05), chlorophyll contents do not differ if they are indicated with the same letter. (B) Chlorophyll contents of infected leaves of WT, lip5-1 and lip5-2 mutant plants complemented with wild-type or mutant LIP5 genes. Pathogen inoculation and chlorophyll content determination were performed as in A. (PDF) [file ppat.1004243.s002.pdf]

Figure S2

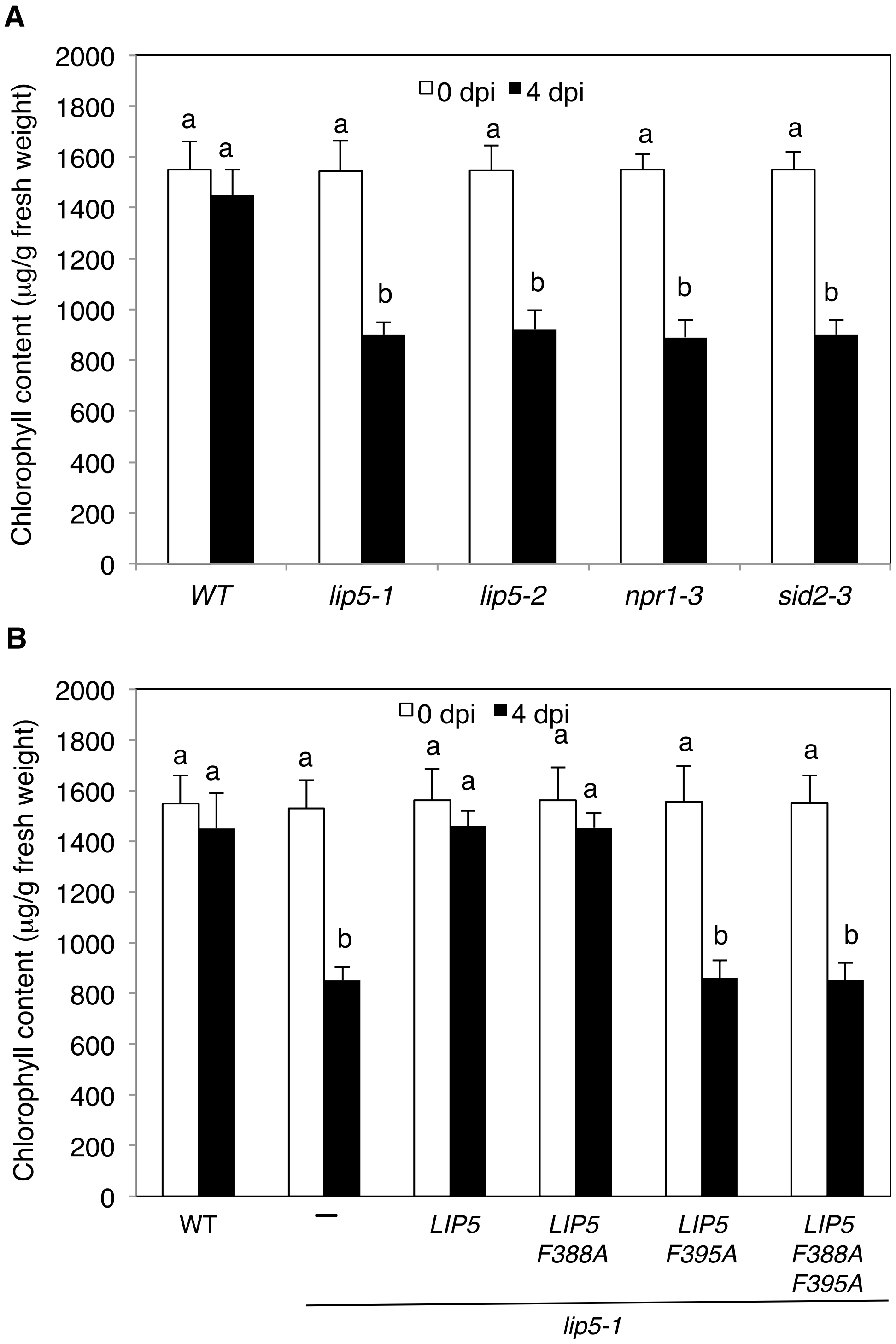
A
Chlorophyll content (μg/g fresh weight)
0 dpi
4 dpi
2000
1800
1600
1400
1200
1000
800
600
400
200
0
a
a
a
b
a
b
a
b
a
b
WT
lip5-1
lip5-2
npr1-3
sid2-3
B
Chlorophyll content (μg/g fresh weight)
0 dpi
4 dpi
2000
1800
1600
1400
1200
1000
800
600
400
200
0
a
a
a
b
a
a
a
a
a
b
a
b
WT
—
LIP5
LIP5 F388A
LIP5 F395A
LIP5 F388A F395A
lip5-1

Figure S2. Effects of *Pst*DC3000 Infection on Chlorophyll Contents of Infected Leaves.

(A) Chlorophyl contents of infected leaves of Wild-type (WT), *lip5, npr1* and *sid2* mutant plants. Plants were infiltrated with a suspension of the virulent *Pst*DC3000 strain (OD_{600} = 0.0002 in 10 mM $MgCl_2$). Samples were taken at 0 and 4 dpi for determination of chlorophyll content. The means and standard errors were calculated from 10 plants for each mutant. According to Duncan's multiple range test (P=0.05), chlorophyll contents do not differ if they are indicated with the same letter.

(B) Chlorophyll contents of infected leaves of WT, *lip5-1* and *lip5-1* mutant plants complemented with wild-type or mutant *LIP5* genes. Pathogen inoculation and chlorophyll content determination were performed as in **A**.
