# Supplementary material for: Arabidopsis LIP5, a Positive Regulator of Multivesicular Body Biogenesis, Is a Critical Target of Pathogen-Responsive MAPK Cascade in Plant Basal Defense
Source: PLoS Pathog. 2014 Jul 10;10(7):e1004243. doi: 10.1371/journal.ppat.1004243 (PMC4092137; doi:10.1371/journal.ppat.1004243)
Supplement: Figure S3 — Responses to avirulent PstDC3000 strains. (A) Pathogen-induced hypersensitive cell death. One half of the wild-type (WT), lip5 and npr1 mutant leaves was infiltrated with a suspension of an avirulent PstDC3000 strain (OD600 = 0.1 in 10 mM MgCl2). Representative inoculated leaves were photographed (upper panel) or subjected to trypan blue staining (lower panel) at the indicated hours post inoculation (hpi). (B) Enhanced susceptibility of the lip5-1 Mutant to avirulent PstDC3000 strains. WT and lip5-1 mutant plants were infiltrated with a suspension of an avirulent PstDC3000 strain (OD600 = 0.0002 in 10 mM MgCl2). Samples were taken at 0 and 5 dpi to determine the growth of the bacterial pathogen. The means and standard errors were calculated from 10 plants for each mutant. According to Duncan's multiple range test (P = 0.05), means of colony-forming units (cfu) do not differ if they are indicated with the same letter. (PDF) [file ppat.1004243.s003.pdf]

Figure S3

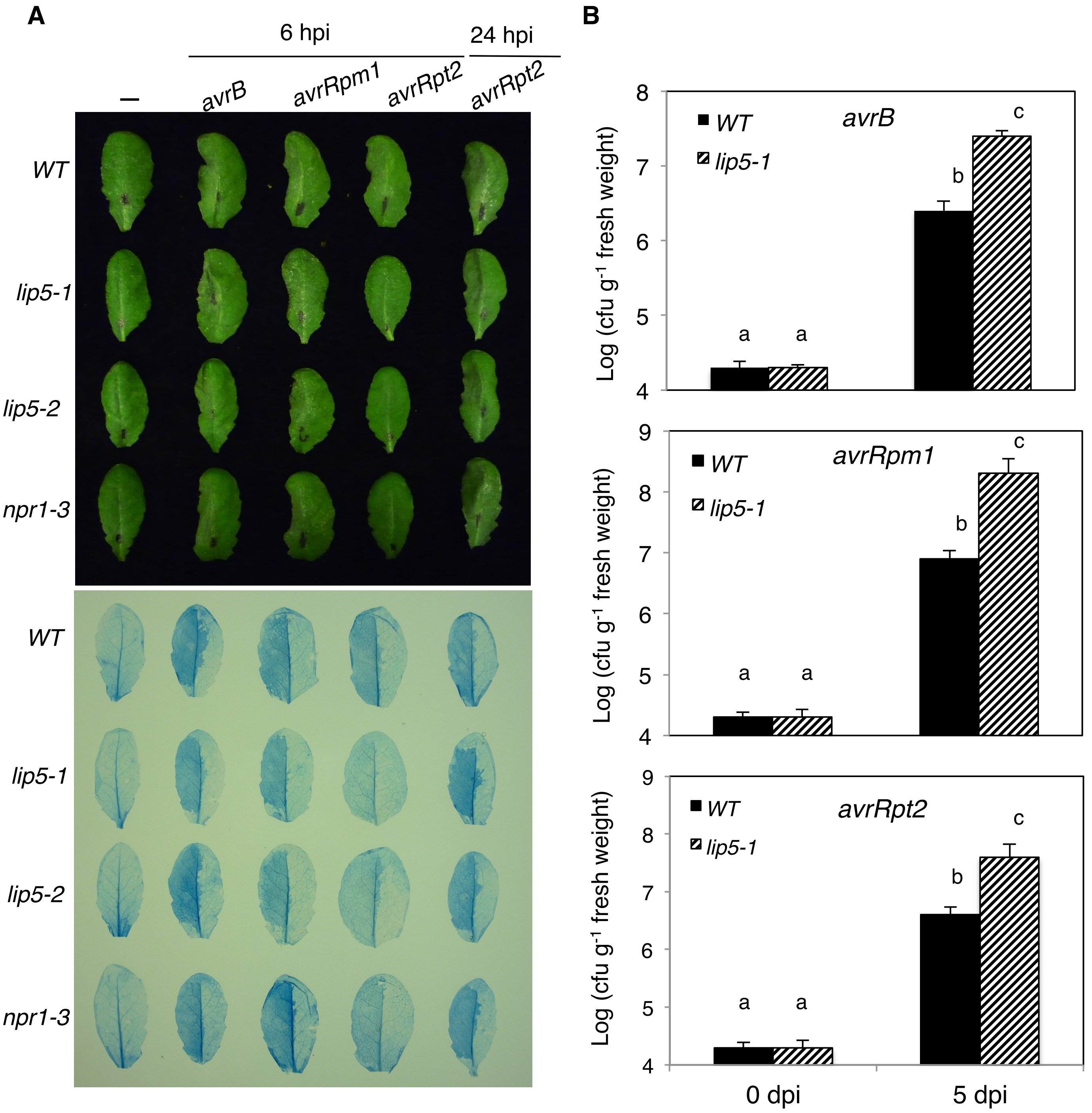
A
6 hpi
24 hpi
—
avrB
avrRpm1
avrRpt2
avrRpt2
WT
lip5-1
lip5-2
npr1-3
WT
lip5-1
lip5-2
npr1-3
B
WT
lip5-1
avrB
avrRpm1
avrRpt2
Log (cfu g-1 fresh weight)
a
b
c
0 dpi
5 dpi

Figure S3. Responses to Avirulent avirulent *Pst*DC3000 strains.

(A) Pathogen-induced hypersensitive cell death. One half of the wild-type (WT), *lip5* and *npr1* mutant leaves was infiltrated with a suspension of an avirulent *Pst*DC3000 strain (OD_{600}=0.1 in 10 mM $MgCl_2$). Representative inoculated leaves were photographed (upper panel) or subjected to trypan blue staining (lower panel) at the indicated hours post inoculation (hpi).

(B) Enhanced susceptibility of the *lip5-1* Mutant to avirulent *Pst*DC3000 strains. WT and *lip5-1* mutant plants were infiltrated with a suspension of an avirulent *Pst*DC3000 strain (OD_{600} = 0.0002 in 10 mM $MgCl_2$). Samples were taken at 0 and 5 dpi to determine the growth of the bacterial pathogen. The means and standard errors were calculated from 10 plants for each mutant. According to Duncan's multiple range test (P=0.05), means of colony-forming units (cfu) do not differ if they are indicated with the same letter.
