# Supplementary material for: Arabidopsis LIP5, a Positive Regulator of Multivesicular Body Biogenesis, Is a Critical Target of Pathogen-Responsive MAPK Cascade in Plant Basal Defense
Source: PLoS Pathog. 2014 Jul 10;10(7):e1004243. doi: 10.1371/journal.ppat.1004243 (PMC4092137; doi:10.1371/journal.ppat.1004243)
Supplement: Figure S4 — LIP5 protein sequences. (A) Alignment of C-terminal domains of LIP5 proteins from yeast, human and Arabidopsis. The conserved tyrosine (Y) and phenylalanine (F) residues critical for interaction with SKD1 are indicated in red. (B) Arabidopsis LIP5 protein sequence. The six putative MPK phosphorylation sites are indicated in red. (PDF) [file ppat.1004243.s004.pdf]

Figure S4

A

```
S. cerevisiae 290   RASKIEQIQKLAKYAISALNYEDLPTAKDELTKALDLLNSI 330
H. sapiens    265   TPEDFARAQKYCKYAGSALQYEDVSTAVQNLQKALKLLTTG 305
A.ahaliana    375   GPEKVAEALKAARFAVGALAFDEVSTAVEHLKKSLELLTNP 415
```

B

```
MSNPNEPARALLPYLQRADELQKHEPLVAYYCRLYAMERGLKIPQSERTKTTNSILMSLINQL
EKDKKSLTLSPDDNMHVEGFALSVFAKADKQDRAGRADLGTAKTFYAASIFFEILSQFGPVPP
DIEQKHKYAAWKAADIRKAIKEGRKPTPGDPVDDDTDLSIPSSGPSGSYDHSASDTNTTSHHR
TELDPPHDSNDDSSHHQFPEVPQHPLPPRFYDNPTNDYPADVPPPPPSSYPSNDHLPPPTGPS
DSPYPHPYSHQPYHQDPPKHMPPPQNYSSHEPSPNSLPNFQSYPSFSESSLPSTSPHYPSHYQ
NPEPYYSSPHSAPAPSSTSFSSAPPPPPYSSNGRINIAPVLDPAPSSAQKYHYDSSYQPGPEK
VAEALKAARFAVGALAFDEVSTAVEHLKKSLELLTNPSAGAGH
```

Figure S4. LIP5 protein sequences.

(A) Alignment of C-terminal domains of LIP5 proteins from yeast, human and Arabidopsis. The conserved tyrosine (Y) and phenylalanine (F) residues critical for interaction with SKD1 are indicated in red.

(B) Arabidopsis LIP5 protein sequence. The six putative MPK phosphorylation sites are indicated in red.
