# Supplementary material for: Arabidopsis LIP5, a Positive Regulator of Multivesicular Body Biogenesis, Is a Critical Target of Pathogen-Responsive MAPK Cascade in Plant Basal Defense
Source: PLoS Pathog. 2014 Jul 10;10(7):e1004243. doi: 10.1371/journal.ppat.1004243 (PMC4092137; doi:10.1371/journal.ppat.1004243)
Supplement: Figure S5 — Yeast two-hybrid assays of mutant LIP5 protein interactions. (A) Yeast two-hybrid assays of LIP5-SKD1 interaction. Full-length LIP5, LIP5F388A, LIP5F395A and LIP5F388A/F395A coding sequences were introduced into the pAD-Gal4 prey vector and cotransformed with the pBD-SKD1 fusion bait vector into yeast cells. Empty pAD-Gal4 vector was used as negative prey control (−). Yeast transformants were analyzed for LacZ reporter gene expression through assays of β-galactosidase activity using ONPG as a substrate. Five separate colonies per construct were used for assays of LacZ β-galactosidase activity. (B) Yeast two-hybrid assays of LIP5 dimerization. Assays with indicated prey and bait vectors were performed as in A. (PDF) [file ppat.1004243.s005.pdf]

Figure S5

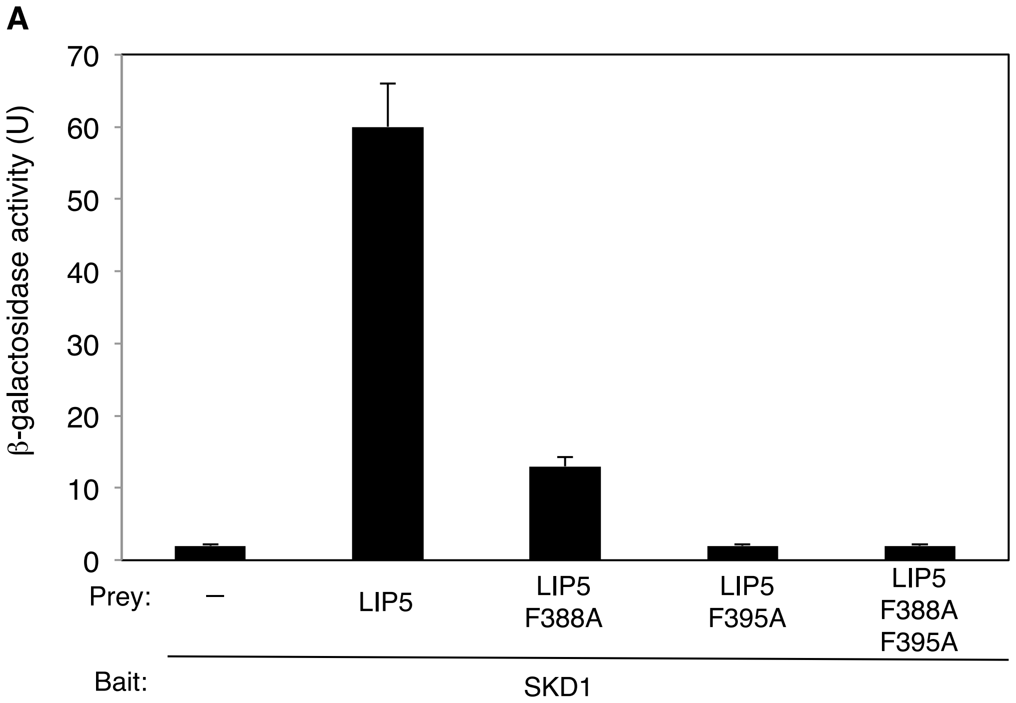
A
β-galactosidase activity (U)
70
60
50
40
30
20
10
0
Prey:
–
LIP5
LIP5
F388A
LIP5
F395A
LIP5
F388A
F395A
Bait:
SKD1

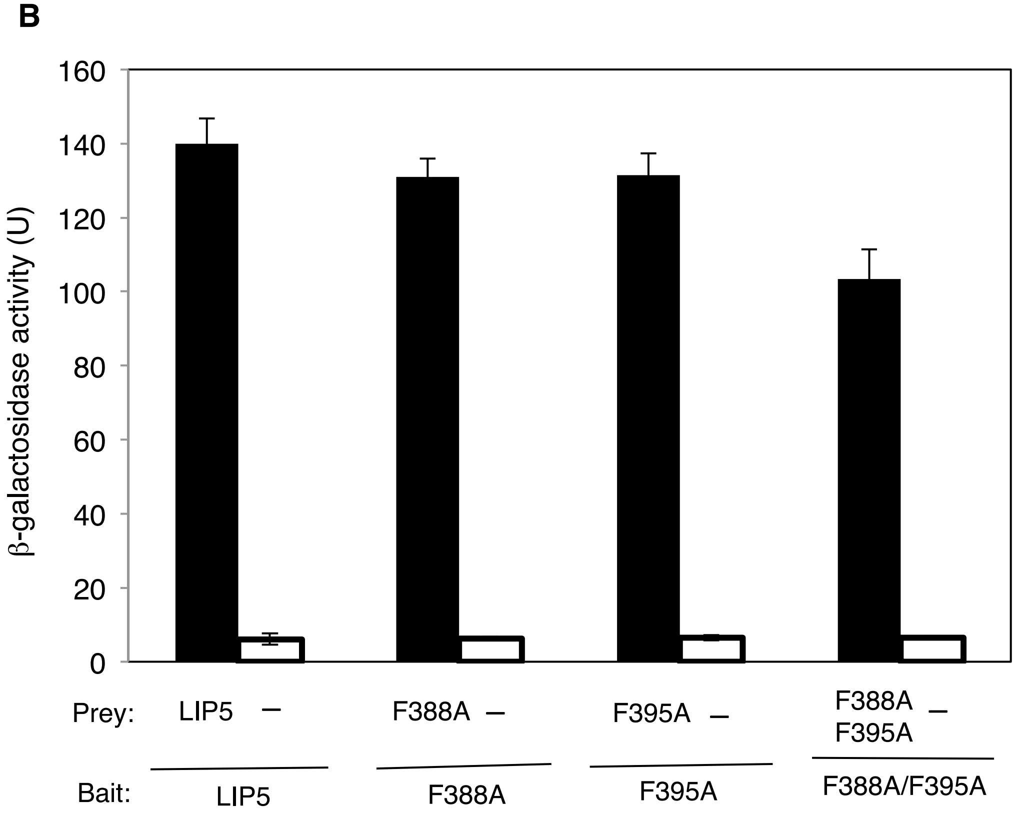
B
β-galactosidase activity (U)
160
140
120
100
80
60
40
20
0
Prey:
LIP5
–
F388A
–
F395A
–
F388A
F395A
–
Bait:
LIP5
F388A
F395A
F388A/F395A

Figure S5. Yeast two-hybrid assays of mutant LIP5 protein interactions.

(A) Yeast two-hybrid assays of LIP5-SKD1 interaction. Full-length *LIP5*, *LIP5F388A*, *LIP5F395A* and *LIP5F388A/F395A* coding sequences were introduced into the pAD-Gal4 prey vector and cotransformed with the pBD-SKD1 fusion bait vector into yeast cells. Empty pAD-Gal4 vector was used as negative prey control (-). Yeast transformants were analyzed for *LacZ* reporter gene expression through assays of β-galactosidase activity using ONPG as a substrate. Five separate colonies per construct were used for assays of LacZ β-galactosidase activity.

(B) Yeast two-hybrid assays of LIP5 dimerization. Assays with indicated prey and bait vectors were performed as in **A**.
