# Supplementary material for: Arabidopsis LIP5, a Positive Regulator of Multivesicular Body Biogenesis, Is a Critical Target of Pathogen-Responsive MAPK Cascade in Plant Basal Defense
Source: PLoS Pathog. 2014 Jul 10;10(7):e1004243. doi: 10.1371/journal.ppat.1004243 (PMC4092137; doi:10.1371/journal.ppat.1004243)
Supplement: Figure S6 — Normal interaction of LIP56A with MPK6 and SKD1 in yeast cells. Full-length LIP56A coding sequence was introduced into the pAD-Gal4 prey vector and was cotransformed with an empty bait vector (−) or with the pBD-MPK6 and pBD-SKD1 fusion bait vectors into yeast cells. Yeast transformants were analyzed for LacZ reporter gene expression through assays of β-galactosidase activity using ONPG as a substrate. Five separate colonies per construct were used for assays of LacZ β-galactosidase activity. (PDF) [file ppat.1004243.s006.pdf]

Figure S6

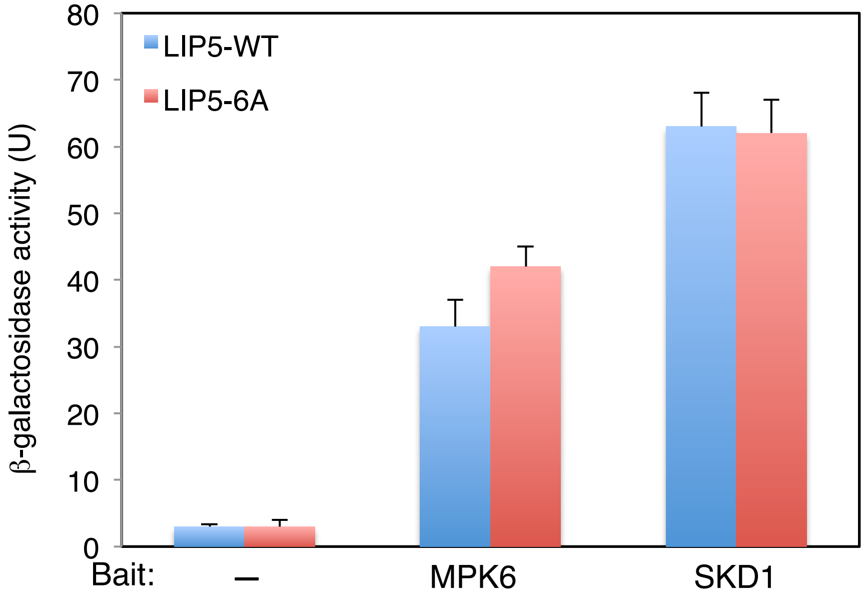

Figure S6. Normal interaction of $LIP5^{6A}$ with MPK6 and SKD1 in yeast cells.

Full-length $LIP5^{6A}$ coding sequence was introduced into the pAD-Gal4 prey vector and was cotransformed with an empty bait vector (-) or with the pBD-MPK6 and pBD-SKD1 fusion bait vectors into yeast cells. Yeast transformants were analyzed for *LacZ* reporter gene expression through assays of β-galactosidase activity using ONPG as a substrate. Five separate colonies per construct were used for assays of LacZ β-galactosidase activity.
