# Supplementary material for: Arabidopsis LIP5, a Positive Regulator of Multivesicular Body Biogenesis, Is a Critical Target of Pathogen-Responsive MAPK Cascade in Plant Basal Defense
Source: PLoS Pathog. 2014 Jul 10;10(7):e1004243. doi: 10.1371/journal.ppat.1004243 (PMC4092137; doi:10.1371/journal.ppat.1004243)
Supplement: Figure S7 — Dephosphorylation of in vivo phosphorylated LIP5 proteins. Protein extracts were isolated from transgenic NtMEK2DD/myc-LIP5WT at 24 hours after DEX treatment (A) or lip5-1/myc-LIP5WT (B) plants at 24 hpi of PstDC3000. The protein extracts was treated at 37°C for 45 minutes with calf intestinal alkaline phosphatase (CIP) in the absence or presence of a phosphatase inhibitor cocktail (10 mM NaF, 7 mM β-glycerophosphate and 5 mM Na-pyrophoshate). Reactions without CIP and phosphatase inhibitors (−) were used as control. The protein extracts were subsequently separated on the regular SDS-PAGE and Phos-tag gels for immunoblot analysis using an anti-myc monoclonal antibody. Rubisco staining of the regular SDS-PAGE gel was used for assessing equal protein loading. (PDF) [file ppat.1004243.s007.pdf]

Figure S7

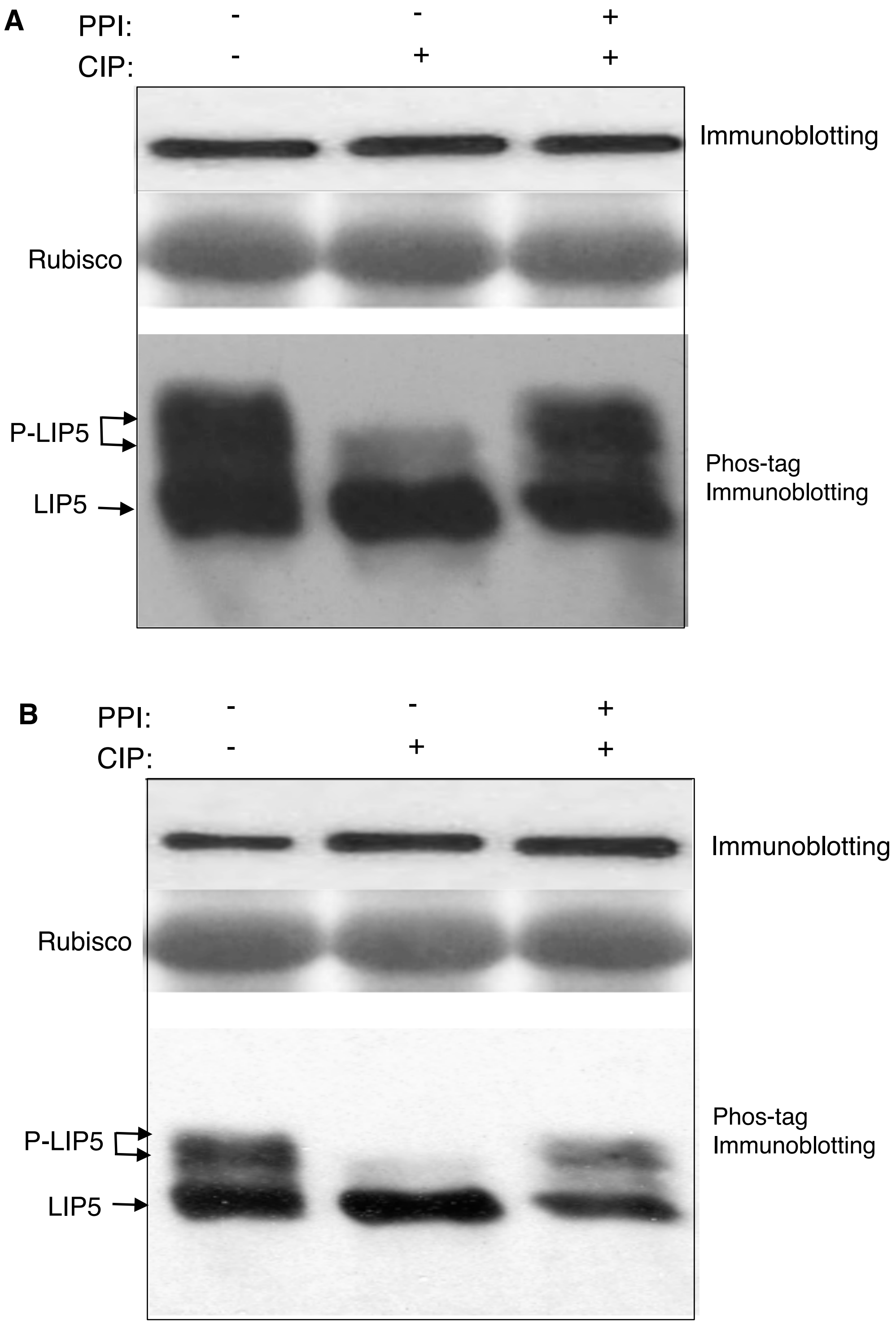
A
PPI: - - +
CIP: - + +
Immunoblotting
Rubisco
P-LIP5
LIP5
Phos-tag
Immunoblotting
B
PPI: - - +
CIP: - + +
Immunoblotting
Rubisco
P-LIP5
LIP5
Phos-tag
Immunoblotting

**Figure S7.** Dephosphorylation of *in vivo* Phosphorylated LIP5 Proteins.

Protein extracts were isolated from transgenic *NtMEK2$^{DD}$/myc-LIP5$^{WT}$* at 24 hours after DEX treatment (**A**) or *lip5-1/myc-LIP5$^{WT}$* (**B**) plants at 24 hpi of *Pst*DC3000. The protein extracts was treated at 37°C for 45 minutes with calf intestinal alkaline phosphatase (CIP) in the absence or presence of a phosphatase inhibitor cocktail (10 mM NaF, 7 mM β-glycerophosphate and 5 mM Na-pyrophoshate). Reactions without CIP and phosphatase inhibitors (-) were used as control. The protein extracts were subsequently separated on the regular SDS-PAGE and Phos-tag gels for immunoblot analysis using an anti-myc monoclonal antibody. Rubisco staining of the regular SDS-PAGE gel was used for assessing equal protein loading.
