# Supplementary material for: Arabidopsis LIP5, a Positive Regulator of Multivesicular Body Biogenesis, Is a Critical Target of Pathogen-Responsive MAPK Cascade in Plant Basal Defense
Source: PLoS Pathog. 2014 Jul 10;10(7):e1004243. doi: 10.1371/journal.ppat.1004243 (PMC4092137; doi:10.1371/journal.ppat.1004243)
Supplement: Figure S8 — Comparison of the transcript levels of the native LIP5 gene and the myc-LIP5 transgenes. Total RNA was isolated from Col-0 wild-type (WT) plants and lip5-1/myc-LIP5WT and lip5-1/myc-LIP56A lines each with similarly high (H), medium (M) and low (L) levels of myc-LIP5 transcripts and probed with a 32P-labeled LIP5 DNA fragment. Ethidium bromide staining of rRNA was shown for the assessment of equal loading. (PDF) [file ppat.1004243.s008.pdf]

Figure S8

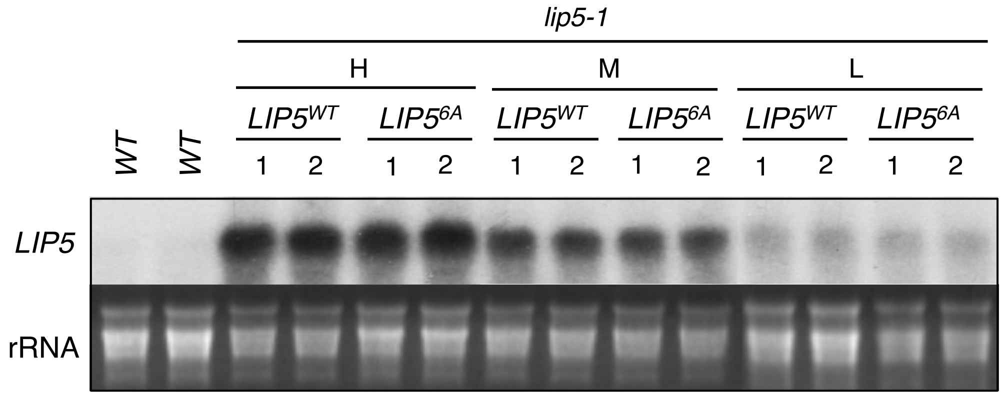

Figure S8. Comparison of the transcript levels of the native *LIP5* gene and the *myc-LIP5* transgenes.

Total RNA was isolated from Col-0 wild-type (WT) plants and *lip5-1/myc-LIP5*^{WT} and *lip5-1/myc-LIP5*^{6A} lines each with similarly high (H), medium (M) and low (L) levels of *myc-LIP5* transcripts and probed with a ^{32}P-labeled *LIP5* DNA fragment. Ethidium bromide staining of rRNA was shown for the assessment of equal loading.
