# Supplementary figures and images for: Arabidopsis LIP5, a Positive Regulator of Multivesicular Body Biogenesis, Is a Critical Target of Pathogen-Responsive MAPK Cascade in Plant Basal Defense
Source: PLoS Pathog. 2014 Jul 10;10(7):e1004243. doi: 10.1371/journal.ppat.1004243 (PMC4092137; doi:10.1371/journal.ppat.1004243)

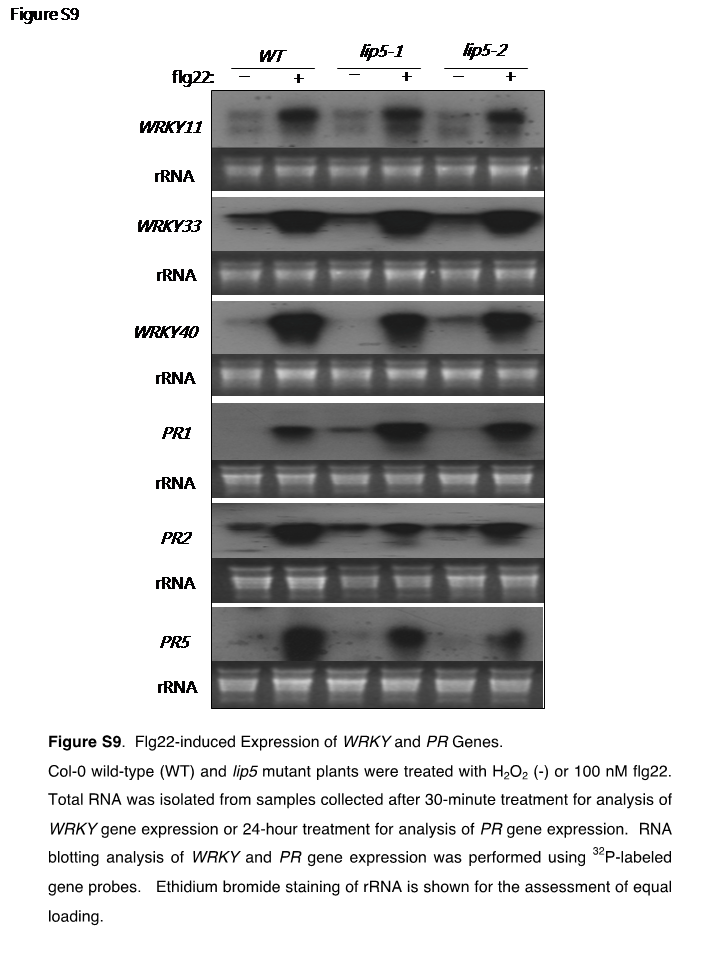

Supplement: Figure S9 — Flg22-induced expression of WRKY and PR genes. Col-0 wild-type (WT) and lip5 mutant plants were treated with H2O2 (−) or 100 nM flg22. Total RNA was isolated from samples collected after 30-minute treatment for analysis of WRKY gene expression or 24-hour treatment for analysis of PR gene expression. RNA blotting analysis of WRKY and PR gene expression was performed using 32P-labeled gene probes. Ethidium bromide staining of rRNA is shown for the assessment of equal loading. (TIF) [file ppat.1004243.s009.tif]
