# Supplementary material for: Arabidopsis LIP5, a Positive Regulator of Multivesicular Body Biogenesis, Is a Critical Target of Pathogen-Responsive MAPK Cascade in Plant Basal Defense
Source: PLoS Pathog. 2014 Jul 10;10(7):e1004243. doi: 10.1371/journal.ppat.1004243 (PMC4092137; doi:10.1371/journal.ppat.1004243)
Supplement: Figure S10 — Expression of LIP5 and SKD1 in response to pathogen infection. Col-0 wild-type plants were infiltrated with 10 mM MgCl2 (mock) or PstDC3000 (OD600 = 0.0002 in 10 mM MgCl2). Samples were collected at indicated days post-inoculation (dpi) for total RNA isolation and RNA blotting analysis of LIP5 and SKD1 gene expression using 32P-labeled gene probes. Ethidium bromide staining of rRNA is shown for the assessment of equal loading. (PDF) [file ppat.1004243.s010.pdf]

Figure S10

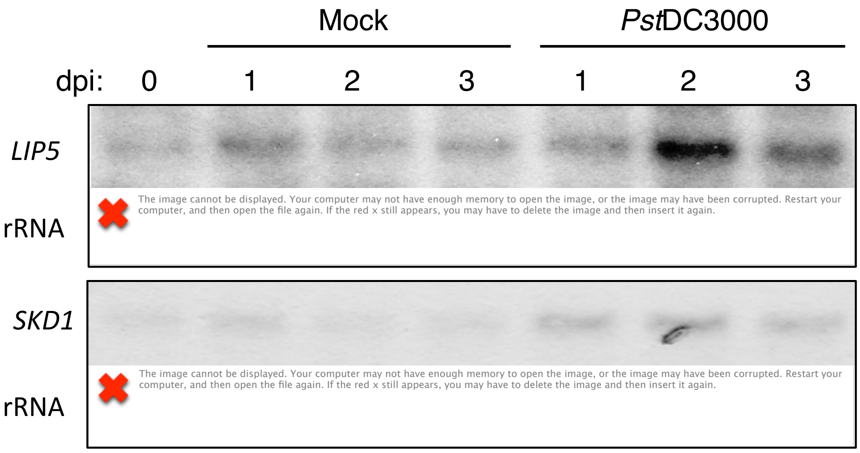

Figure S10. Expression of *LIP5* and *SKD1* in Response to Pathogen Infection.

Col-0 wild-type plants were infiltrated with 10 mM $MgCl_2$ (mock) or *Pst*DC3000 (OD_{600}=0.0002 in 10 mM $MgCl_2$). Samples were collected at indicated days post-inoculation (dpi) for total RNA isolation and RNA blotting analysis of *LIP5* and *SKD1* gene expression using ^{32}P-labeled gene probes. Ethidium bromide staining of rRNA is shown for the assessment of equal loading.
