# Supplementary material for: Arabidopsis LIP5, a Positive Regulator of Multivesicular Body Biogenesis, Is a Critical Target of Pathogen-Responsive MAPK Cascade in Plant Basal Defense
Source: PLoS Pathog. 2014 Jul 10;10(7):e1004243. doi: 10.1371/journal.ppat.1004243 (PMC4092137; doi:10.1371/journal.ppat.1004243)
Supplement: Figure S11 — Activity of LIP5-GFP in plant disease resistance. (A) Western blotting analysis of LIP5-GFP fusion protein. Total proteins were isolated from untransformed Arabidopsis (−) or transgenic LIP5-GFP plants and subjected to western blot analysis using an anti-GFP monoclonal antibody. The antibody detected a protein band with molecular mass expected to be that of LIP5-GFP from transgenic LIP5-GFP plants. Other proteins detected by the antibody due to nonspecific binding are present in both untransformed Arabidopsis and transgenic LIP5-GFP plants. (B) Disease symptom development. Wild type (WT), lip5-1 and lip5-1/LIP5-GFP plants were infiltrated with a suspension of PstDC3000 (OD600 = 0.0002 in 10 mM MgCl2). Pictures of representative inoculated leaves taken at 4 dpi. (C) Bacterial growth. Pathogen inoculation of wild-type and mutant plants was performed as in A. Samples were taken at 0 or 4 dpi to determine the bacterial growth. The means and standard errors were calculated from 10 plants for each mutant. According to Duncan's multiple range test (P = 0.05), means of colony-forming units (cfu) do not differ if they are indicated with the same letter. (PDF) [file ppat.1004243.s011.pdf]

Figure S11

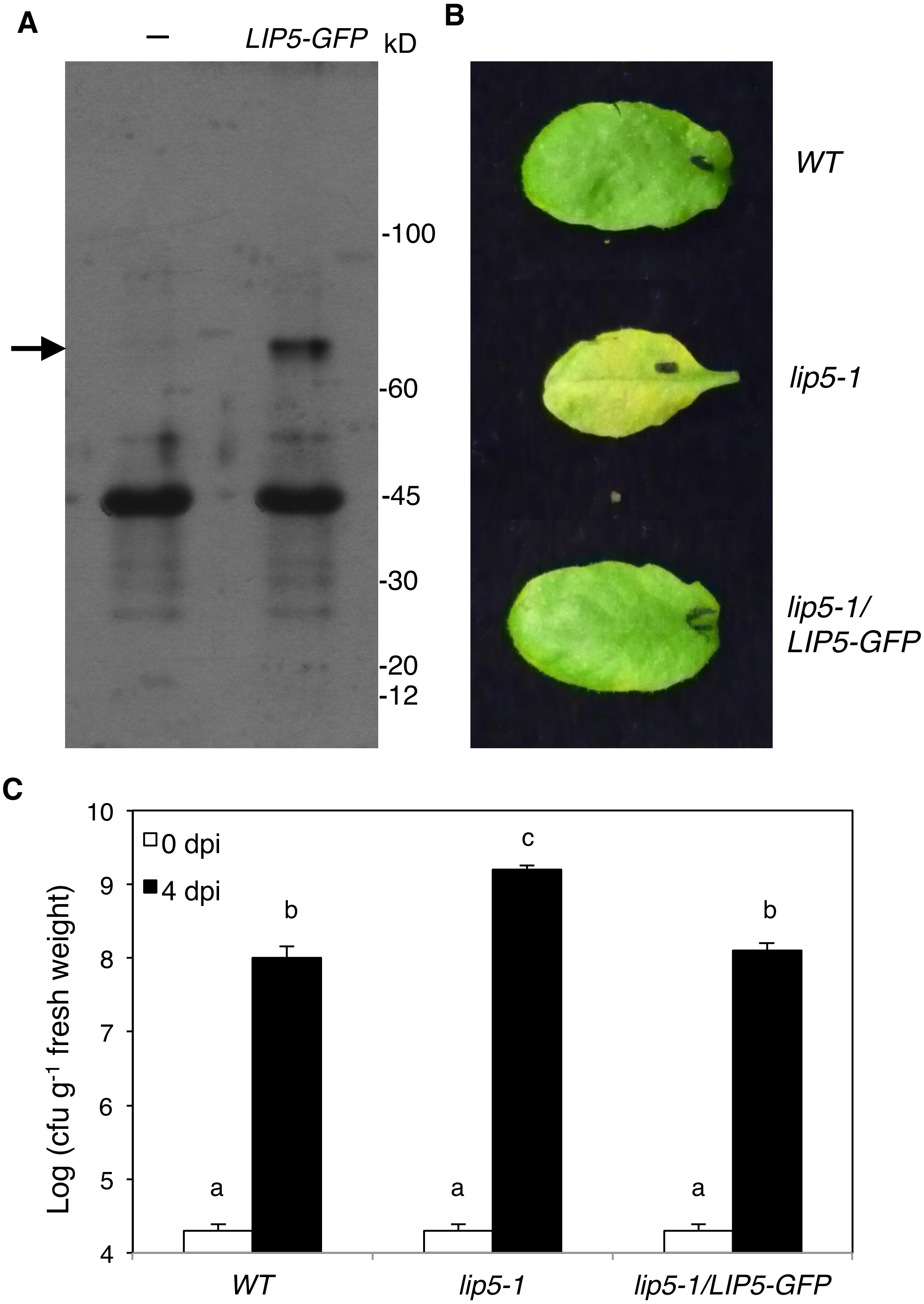
A
—
LIP5-GFP
kD
-100
-60
-45
-30
-20
-12
B
WT
lip5-1
lip5-1/
LIP5-GFP
C
Log (cfu g-1 fresh weight)
0 dpi
4 dpi
10
9
8
7
6
5
4
a
b
a
c
a
b
WT
lip5-1
lip5-1/LIP5-GFP

Figure S11. Activity of LIP5-GFP in plant disease resistance.

(A) Western blotting analysis of LIP5-GFP fusion protein. Total proteins were isolated from untransformed Arabidopsis (-) or transgenic LIP5-GFP plants and subjected to western blot analysis using an anti-GFP monoclonal antibody. The antibody detected a protein band with molecular mass expected to be that of LIP5-GFP from transgenic LIP5-GFP plants. Other proteins detected by the antibody due to nonspecific binding are present in both untransformed Arabidopsis and transgenic *LIP5-GFP* plants.

(B) Disease symptom development. Wild type (WT), *lip5-1* and *lip5-1/LIP5-GFP* plants were infiltrated with a suspension of *Pst*DC3000 (OD_{600}=0.0002 in 10 mM $MgCl_2$). Pictures of representative inoculated leaves taken at 4 dpi.

(C) Bacterial growth. Pathogen inoculation of wild-type and mutant plants was performed as in **A.** Samples were taken at 0 or 4 dpi to determine the bacterial growth. The means and standard errors were calculated from 10 plants for each mutant. According to Duncan's multiple range test (P=0.05), means of colony-forming units (cfu) do not differ if they are indicated with the same letter.
