# Supplementary material for: Arabidopsis LIP5, a Positive Regulator of Multivesicular Body Biogenesis, Is a Critical Target of Pathogen-Responsive MAPK Cascade in Plant Basal Defense
Source: PLoS Pathog. 2014 Jul 10;10(7):e1004243. doi: 10.1371/journal.ppat.1004243 (PMC4092137; doi:10.1371/journal.ppat.1004243)
Supplement: Figure S12 — Wound-induced alteration in subcellular localization of LIP5-GFP in leaf epidermal cells. Leaf epidermal layer of transgenic Arabidopsis plants expressing LIP5-GFP was peeled off, stained in a DAPI solution and observed under a confocal microscope. Bars = 10 µm. (PDF) [file ppat.1004243.s012.pdf]

Figure S12

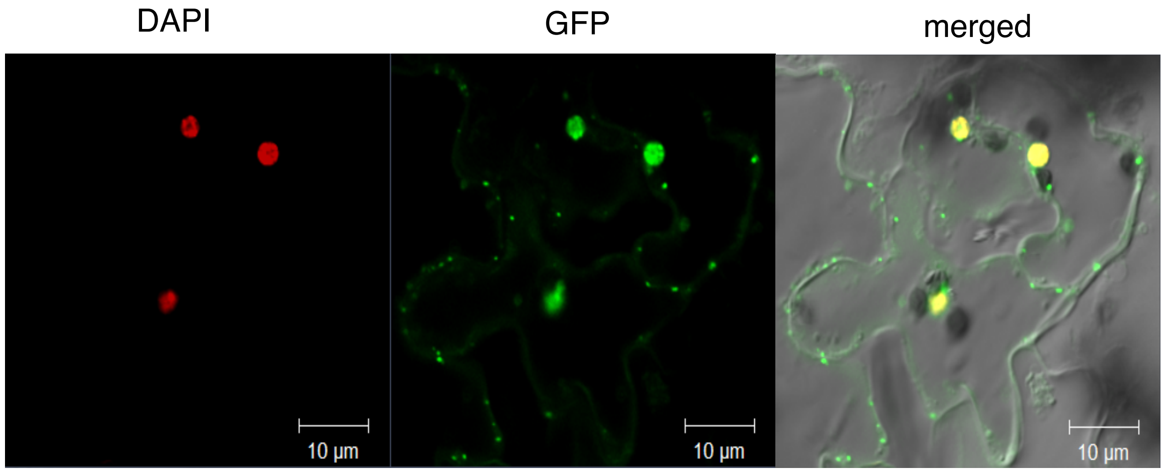

Figure S12. Wound-induced alteration in subcellular localization of LIP5-GFP in leaf epidermal cells.

Leaf epidermal layer of transgenic Arabidopsis plants expressing *LIP5-GFP* was peeled off, stained in a DAPI solution and observed under a confocal microscope. Bars =10 μm
