# Supplementary material for: Arabidopsis LIP5, a Positive Regulator of Multivesicular Body Biogenesis, Is a Critical Target of Pathogen-Responsive MAPK Cascade in Plant Basal Defense
Source: PLoS Pathog. 2014 Jul 10;10(7):e1004243. doi: 10.1371/journal.ppat.1004243 (PMC4092137; doi:10.1371/journal.ppat.1004243)
Supplement: Table S1 — Primers for screening of T-DNA insertion mutants. (PDF) [file ppat.1004243.s013.pdf]

Table S1. Primers for screening of T-DNA insertion mutants.

MUTANT	PRIMERS	
	FORWARD	REVERSE
lip5-1	attccctgaagtgccacaac	aacatcaaccccaagtgtca
lip5-2	aggaagcccgctccaggtgatcc	gcagagtgcggagcagaatagta
sid2-3	caatccgatttgctgctgta	gcatggccacactaactaaaa
fls2-2	cgggaaaataccagaatgctt	tagatccgccgggagctca
mpk3	gtgcagttgaacaagctctg	tagacaactcacggcgacag
mpk6	gctgatgcaaaattgtttagg	gaagagtggcttacggtcca
