# Supplementary material for: Arabidopsis LIP5, a Positive Regulator of Multivesicular Body Biogenesis, Is a Critical Target of Pathogen-Responsive MAPK Cascade in Plant Basal Defense
Source: PLoS Pathog. 2014 Jul 10;10(7):e1004243. doi: 10.1371/journal.ppat.1004243 (PMC4092137; doi:10.1371/journal.ppat.1004243)
Supplement: Table S2 — Primers for construct mutant LIP5 and SKD1 genes. (PDF) [file ppat.1004243.s014.pdf]

Table S2. Primers for construct mutant *LIP5* and *SKD1* genes.

MUTANT GENE	PRIMERS
LIP5F388A	F: gcactcaaggctgctagagccgctgtgggagctttgg<br>R: ccaaagctcccacagcggctctagcagccttgagtgc
LIP5F395A	F: tgtgggagctttggctgctgatgaagtct<br>R: agacttcatcagcagccaaagctcccaca
LIP5S73A	F: cattgactctggccccagatgac<br>R: gtcatctggggccagagtcaatg
LIP5T153A	F: aggaagcccgctccaggtgatcc<br>R: ggatcacctggagcgggcttcct
LIP5S254A	F: ccatcagacgccccttacccgca<br>R: tgcgggtaaggggcgtctgatgg
LIP5S285A	F: tgagcctgctccaaattctctcc<br>R: ggagagaatttggagcaggctca
LIP5S307A	F: tcccatccactgctccccactac<br>R: gtagtggggagcagtggatggga
LIP5S323A	F: tactattctgctccgcactctgc<br>R: gcagagtgcggagcagaatagta
SKD1E232Q	F: accacacaaagaatctatctgatcaacaaaaataa<br>R: cctcgattatttttgttgatcagatagattctttg
